# Hierarchical routing in carbon metabolism favors iron-scavenging strategy in iron-deficient soil *Pseudomonas* species

**DOI:** 10.1073/pnas.2016380117

**Published:** 2020-12-03

**Authors:** Caroll M. Mendonca, Sho Yoshitake, Hua Wei, Anne Werner, Samantha S. Sasnow, Theodore W. Thannhauser, Ludmilla Aristilde

**Affiliations:** ^a^Department of Biological and Environmental Engineering, College of Agriculture and Life Sciences, Cornell University, Ithaca, NY 14853;; ^b^Department of Civil and Environmental Engineering, McCormick School of Engineering and Applied Science, Northwestern University, Evanston, IL 60208;; ^c^Robert W. Holley Center for Agriculture and Health, United States Department of Agriculture, Agricultural Research Service, Cornell University, Ithaca, NY 14853

**Keywords:** metabolomics, iron limitation, siderophore, bacteria, *Pseudomonas putida*

## Abstract

High-affinity iron (Fe) scavenging compounds, or siderophores, are widely employed by soil bacteria to survive scarcity in bioavailable Fe. Siderophore biosynthesis relies on cellular carbon metabolism, despite reported decrease in both carbon uptake and Fe-containing metabolic proteins in Fe-deficient cells. Given this paradox, the metabolic network required to sustain the Fe-scavenging strategy is poorly understood. Here, through multiple ^13^C-metabolomics experiments with Fe-replete and Fe-limited cells, we uncover how soil *Pseudomonas* species reprogram their metabolic pathways to prioritize siderophore biosynthesis. Across the three species investigated (*Pseudomonas putida* KT2440, *Pseudomonas protegens* Pf-5, and *Pseudomonas putida* S12), siderophore secretion is higher during growth on gluconeogenic substrates than during growth on glycolytic substrates. In response to Fe limitation, we capture decreased flux toward the tricarboxylic acid (TCA) cycle during the metabolism of glycolytic substrates but, due to carbon recycling to the TCA cycle via enhanced anaplerosis, the metabolism of gluconeogenic substrates results in an increase in both siderophore secretion (up to threefold) and Fe extraction (up to sixfold) from soil minerals. During simultaneous feeding on the different substrate types, Fe deficiency triggers a hierarchy in substrate utilization, which is facilitated by changes in protein abundances for substrate uptake and initial catabolism. Rerouted metabolism further promotes favorable fluxes in the TCA cycle and the gluconeogenesis–anaplerosis nodes, despite decrease in several proteins in these pathways, to meet carbon and energy demands for siderophore precursors in accordance with increased proteins for siderophore biosynthesis. Hierarchical carbon metabolism thus serves as a critical survival strategy during the metal nutrient deficiency.

## Significance

Siderophore secretion confers competitive advantage to pathogenic and beneficial bacteria in various nutritional environments, including human infections and rhizosphere microbiome. The siderophore biosynthesis must be sustained during a compromised carbon metabolism in Fe-deficient cells. Here we demonstrate that Fe-deficient *Pseudomonas* species overcome this paradox by coupling selectivity in carbon utilization with a hierarchy in metabolic pathways to favor carbon and energy fluxes for siderophore biosynthesis. A reprogrammed metabolism is predicted from genomics-based data obtained with several marine and soil bacterial systems in response to Fe deficiency, but metabolomics evidence is lacking. The present study offers an important roadmap for investigating the underlying metabolic connections between Fe or other metal nutrient availability and carbon utilization.

**Fig. 1.**
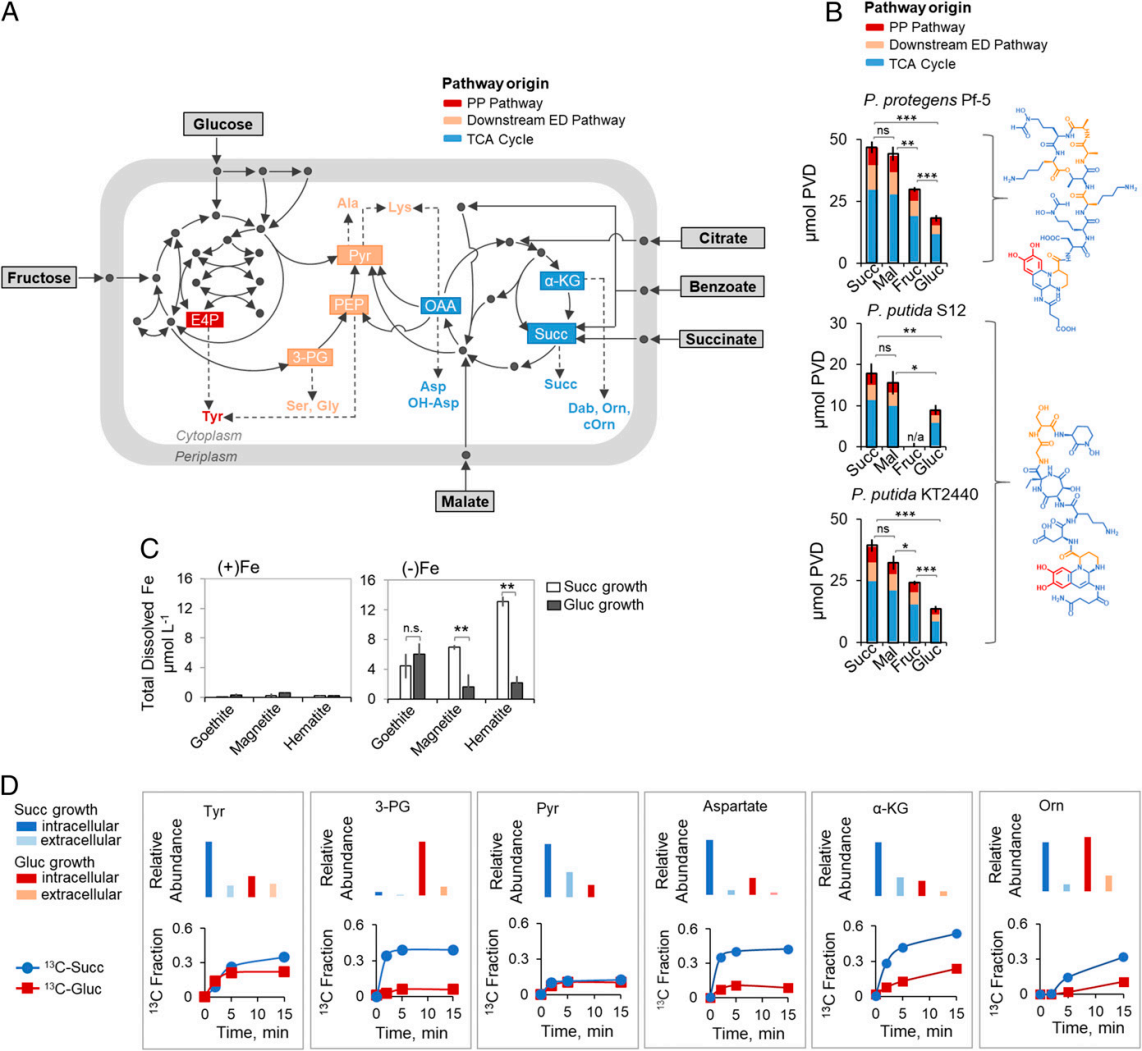
Gluconeogenic substrates lead to higher siderophore yield and Fe scavenging relative to glycolytic substrates in *Pseudomonas* spp. (*A*) Schematic metabolic routing of gluconeogenic substrates (succinate, malate, and benzoate) and glycolytic substrates (glucose and fructose) toward metabolite precursors for PVD biosynthesis in *Pseudomonas* species. (*B*) Substrate-dependent PVD concentration (mean ± SD µmol), normalized by biomass at the time of sampling, produced by Fe-limited *P. protegens* Pf-5, *P. putida* S12, and *P. putida* KT2440 following growth on carbon-equivalent (100 mM C) succinate, malate, fructose, or glucose as the sole carbon source; the chemical structure of the primary PVD produced by each species is shown on the *Right* (one-way ANOVA, Tukey’s studentized range test: ns, not statistically significant; *P < 0.05; **P < 0.01; ***P < 0.001). (*C*) Total dissolved Fe (mean ± SD µmol L^−1^) from the dissolution of Fe-bearing minerals (goethite, magnetite, and hematite; 1 g L^−1^) reacted with bacterial secretions obtained with intermediate Fe-limited *P. putida* KT2440 cells following growth on glucose or succinate (two-tailed *t* test: **P < 0.01, ns, not significant). (*D*) Relative abundance (*Top*) and kinetic ^13^C profiling (*Bottom*) of PVD metabolite precursors (from *Left* to *Right*: tyrosine, 3-PG, pyruvate, aspartate, α-KG, and Orn) in intermediate Fe-limited *P. putida* KT2440 cells fed on 50:50 [U-^13^C_6_]-glucose:unlabeled glucose (red symbols) or 50:50 [U-^13^C_4_]-succinate:unlabeled succinate (blue symbols); in both sets of data, error bars are too small to be noticed beyond the data points of averaged values from the biological replicates. Color code for pathway designation in *A* and *B*: PP pathway in red, downstream ED pathway in light orange, and TCA cycle in blue. Metabolite and substrate abbreviations for *A*–*D*: succinate, Succ; malate, Mal; glucose, Gluc; fructose, Fruc; erythrose 4-phosphate, E4P; 3-phosphoglycerate, 3-PG; phosphoenolpyruvate, PEP; pyruvate, Pyr; oxaloacetate, OAA; α-ketoglutarate, α-KG; tyrosine, Tyr; serine, Ser; glycine, Gly; alanine, Ala; lysine, Lys; aspartate, Asp; hydroxyl-aspartate, OH-Asp; diamino butyrate, Dab, ornithine, Orn; cyclic ornithine, cOrn. All data are from three biological replicates.

The low solubility of iron (Fe) oxides and hydroxides, in addition to strong organic complexes of Fe, limits the bioavailability of Fe in aerobic environments (1–4). However, except in some ecological adaptations, ample Fe supply is needed to sustain optimal cellular functions in aerobic bacteria due to the requirement of hemes and Fe-sulfur cofactors (3, 5, 6). Siderophore secretion for Fe acquisition is one notable strategy implicated in conferring nutritional advantage to aerobic bacteria, including *Pseudomonas* species (6–8). The primary siderophores secreted by these species are pyoverdines (PVDs), which are nonribosomal peptidic compounds synthesized directly from metabolites from the central carbon metabolism (7) (Fig. 1*A*). To a lesser extent, *Pseudomonas* species also secrete other siderophores such as pyochelin and organic acids, both of which have lower Fe-binding affinity (7, 9). The PVD biosynthesis requires carbon investment from several pathways in the central carbon metabolism (Fig. 1*A*). The PVD structures contain, in addition to a peptide chain, a 2,3-diamino-6,7-dihydroxyquinoline chromophore and a side chain (7, 10) (Fig. 1 *A* and *B*). The PVD peptide chain, which contains amino acids primarily derived from lower glycolysis and the tricarboxylic acid (TCA) cycle, differs among species within the *Pseudomonas* genus by the amount, sequence, and configuration of the amino acids (7) (Fig. 1*B*). The Fe-binding catecholate and hydroxamate moieties are synthesized from the pentose-phosphate (PP) pathway and TCA cycle metabolites: the catecholate moiety, derived from chorismate, is synthesized from erythrose-4-phosphate (E4P, a PP pathway metabolite) and phosphoenolpyruvate (PEP, a metabolite upstream of the TCA cycle); the hydroxamate moiety is derived from α-ketoglutarate (α-KG, a TCA cycle metabolite) (7, 10, 11) (Fig. 1*A*). The side chain, which is a dicarboxylic acid or its monoamide derivative, is derived from the TCA cycle (7, 10, 11) (Fig. 1*A*). In addition, seven moles of acetyl-CoA are needed to compose the fatty acid chain attached to the pre-PVD structure before translocation of the final structure from the cytosol to the periplasm (12). Thus, the PVD biosynthesis is a carbon-expensive endeavor that consumes metabolic precursors away from biomass growth. In fact, decrease in both biomass growth and carbon assimilation has been reported for various Fe-limited organisms such as the soil bacterium *Pseudomonas putida* (9), the marine diatom *Chaetoceros brevis* (13), and the cynaobacterium *Synechococcus* sp. PCC7002 (14). For *Pseudomonas* species, Fe-limited cells have to cope with both the metabolic burden of the PVD siderophore biosynthesis and compromised carbon metabolism.

Genome- and transcriptome-level regulations of PVD biosynthesis have been studied in several important *Pseudomonas* species, including crop-beneficial (*P. putida*, *Pseudomonas* *fluorescens*, and *Pseudomonas protegens*) (6, 15), plant pathogenic (*Pseudomonas syringae*) (16, 17), and human pathogenic (*Pseudomonas aeruginosa*) species (7, 18). In *P. aeruginosa* involved in cystic fibrosis infections, multiple Fe-uptake genes were activated (19) and expression of PVD-related genes was induced (18, 20) to support this bacterial pathogenesis (21–23). For the plant pathogen *P. syringae*, decreased expression of virulence genes in Fe-limited cells also highlighted the importance of Fe supply for this pathogenic species (16). For plant-beneficial *P. fluorescens*, both genes and proteins for siderophore transport and biosynthesis were increased in response to Fe limitation (6). Along with this gene-level regulation in *P. fluorescens*, carbon source was shown to influence the rate of PVD production, such that 40% more PVD was produced by succinate-grown cells than by glucose-grown cells (24).

Little is known, however, about how Fe-limited cells manage their intracellular carbon metabolism to sustain siderophore biosynthesis. Decrease in the gene expression or protein abundance of one or both of the Fe-containing metabolic enzymes (namely, aconitate hydratase and succinate dehydrogenase) in the TCA cycle has been reported in several Fe-limited bacterial species [*P. protegens* (6), *Alteromonas macleodii* (25), and *Escherichia coli* (5)] and in Fe-limited yeast [*Saccharomyces cerevisiae* (26)]. Changes in other TCA cycle enzymes were also reported, including decrease in the gene expression and levels of fumarate hydratase in *P. protegens* (6), decreased abundance of citrate synthase and succinyl-CoA synthetase in *E. coli* (5), and increase in both expression and levels of isocitrate lyase in *Pseudomonas angustum* (27). Other reported strategies to overcome stress from Fe limitation included induction of the glyoxylate shunt in the TCA cycle of *P. angustum* (27), changes in the amount and diversity of carbon utilization in *Fusobacterium* (28), and rerouting of substrate catabolism via overflow metabolism in *P. putida* (9), *E. coli* (5), and *Caballeronia mineralivorans* (29). Despite the reported decrease in gene expression or enzyme abundance for several metabolic proteins as well as changes in carbon usage, the consequences on the metabolic network fluxes required for the siderophore biosynthetic fluxes have remained unknown.

Here three hypotheses were tested in regards to the connections between Fe-limited carbon metabolism and PVD biosynthesis in *Pseudomonas* species: first, that the siderophore yield would be higher from gluconeogenic substrates than from glycolytic substrates due to relatively higher carbon fluxes to the TCA cycle from the gluconeogenic substrates; second, that Fe-limited cellular metabolism during growth on a substrate mixture would be programmed for selective carbon utilization toward the TCA cycle to provide for siderophore precursors; and, third, that changes in the metabolic network would serve to promote specific carbon fluxes toward overcoming depletion of Fe-containing metabolic enzymes. To test these hypotheses, we applied stable isotope-assisted metabolomics approaches coupled with proteomics profiling with cells grown under different carbon sources and Fe levels. We obtained multiple dynamic and long-term ^13^C metabolite labeling to track Fe-dependent assimilation of different types of ^13^C-labeled substrates into metabolic pathways and subsequently determined the partitioning of fluxes in cellular metabolism in response to Fe deficiency. To evaluate changes in protein levels as determinants of metabolic flux changes, we profiled the abundance of proteins in central carbon metabolism as well as proteins involved in Fe transport and regulation and siderophore biosynthesis.

## Results

Experiments (in three biological replicates) were performed with the following soil *Pseudomonas* species: *P. protegens* Pf-5, *P. putida* S12, or *P. putida* KT2440. As a plant-beneficial rhizosphere isolate, *P. protegens* Pf-5 (formerly identified as *P. fluorescens* Pf-5) is known to produce nutrient-scavenging compounds and antimicrobials toxic to plant pathogens (6, 30, 31). Both *P. putida* S12 and *P. putida* KT2440 are model soil species widely employed as biocatalytic platforms due to their metabolic versatility (32, 33). We acclimated the cells to different Fe conditions and carbon substrates. The Fe conditions, which were characterized by computing the total unchelated Fe concentration (i.e., unchelated by disodium ethylene-diamine-tetra-acetic-acid [EDTA], a strong Fe chelator) in the nutrient composition, were Fe-replete (with 30 μM unchelated Fe), intermediate Fe-limited (with 650 nM unchelated Fe), and Fe-limited (38 nM unchelated Fe) conditions (see *SI Appendix*, Table S1 and *Methods* for more details). The carbon source, with a total carbon-equivalent concentration of 100 mM and 300 mM to ensure optimal carbon supply for biomass growth (34) when Fe supply is limited, was provided either as a single substrate or as an equimolar carbon mixture of two substrates. Single-substrate growth was with glucose, fructose, malate, succinate, or citrate; mixed-substrate growth involved a glucose:succinate, glucose:citrate, glucose:acetate, or glucose:benzoate mixture. Glucose and fructose, which are common monosaccharides in plant secretions and in the soil matrix (35), represent glycolytic substrates that undergo glycolysis through the Entner–Doudoroff (ED) pathway in *Pseudomonas* species before feeding the TCA cycle through two pyruvate compounds (9, 36, 37) (Fig. 1*A*). As abundant metabolic organic acid products from microorganisms and plant roots (38), succinate, malate, acetate are gluconeogenic substrates that enter metabolism directly via TCA cycle intermediates (Fig. 1*A*). Benzoate, which is a derivative of lignin-compounds as well as an intermediate in the catabolism of other aromatic compounds (39), was chosen as both a poor Fe chelator and a gluconeogenic substrate. In *P. putida* KT2440 cells, benzoate-derived carbons enter central carbon metabolism via one mole of succinate and one mole of acetyl-CoA, both of which feed directly the TCA cycle (Fig. 1*A*).

### Higher Siderophore Production and Fe Scavenging with Gluconeogenic Substrates Than with Glycolytic Substrates.

We investigated substrate- and species-dependent PVD production by the three *Pseudomonas* species following growth on four substrates: two carbohydrates (glucose and fructose) and two short-chain carboxylic acids (succinate and malate) (Fig. 1*B*). Acknowledging that multiple PVD structures may be secreted, in addition to the reported primary structure for each species, we monitored the chromophore common to all PVDs to quantify the total PVD concentrations (10) (*SI Appendix*, Fig. S1). Across all three strains, the biomass-normalized PVD concentrations were 34% to threefold higher following growth on the gluconeogenic substrates than following growth on the glycolytic substrates (Fig. 1*B*).

Based on the structural composition of the primary PVD structures, we estimated the relative contribution of different metabolic pathways for each PVD biosynthesis (Fig. 1*B*). The two *P. putida* strains, *P. putida* KT2440 and *P. putida* S12, have reportedly identical peptides of seven amino acids (aspartate-ornithine-hydroxyaspartate-diaminobutyrate-glycine-serine-cyclic hydroxyornithine) in their primary PVD structures (10, 40) but the *P. protegens* strain has a different peptide of eight amino acids of different composition (aspartatate-formylhydroxyornithine-lysine-threonine-alanine-alanine-formylhydroxyornithine-lysine) (40, 41) in its primary PVD structure (Fig. 1*B*). For these PVD structures, the highest carbon demand was from the TCA cycle (58 to 63%), followed by metabolites from downstream of the ED pathway (32 to 34%) and from the PP pathway (8 to 9%) (Fig. 1*B*). Therefore, PVD biosynthesis requires significant carbon investment from the TCA cycle to meet the carbon demands primarily from α-KG, followed by oxaloacetate (OAA) and succinate (Fig. 1*B* and *SI Appendix*, Fig. S2*A*).

Our data implied that the metabolic burden of PVD biosynthesis may be responsible for the preference of gluconeogenic substrates over the glycolytic substrates to favor PVD production by the Fe-limited *Pseudomonas* species. Furthermore, we found that the PVD-containing bacterial secretions resulted in 4.4 to 13.1 µM dissolved Fe (on average) following reactions with three crystalline Fe-bearing oxide minerals (goethite, hematite, and magnetite; 1 g L^−1^) (Fig. 1*C*). Notably, relative to the glucose-derived bacterial secretions, the succinate-derived bacterial secretions led to up to sixfold higher dissolved Fe concentration from hematite and magnetite (Fig. 1*C*). Therefore, the enhanced secretion of PVD from gluconeogenic substrates was advantageous to Fe scavenging (Fig. 1*C*).

### Increased Fluxes through the TCA Cycle Are Responsible for Enhanced Siderophore Production.

We performed kinetic isotopic profiling of *P. putida* KT2440 fed on [U-^13^C_4_]-succinate or on [U-^13^C_6_]-glucose to capture in vivo carbon fluxes into six PVD precursors: tyrosine, 3-phosphoglycerate (3-PG), pyruvate, aspartate, α-KG, and ornithine; we also obtained relative intracellular and extracellular levels of each metabolite (Fig. 1*D*). Compared to feeding on the labeled glucose, feeding on the labeled succinate resulted in a two- to fivefold higher isotopic enrichment in four of the metabolites (3-PG, aspartate, α-KG, and ornithine), three of which are derived from the TCA cycle (Fig. 1 *A* and *D*). The ^13^C enrichment of tyrosine and pyruvate was the same during feeding on both substrates (Fig. 1*D*). Tyrosine is made from metabolites derived from both the PP pathway and downstream of the ED pathway, which may explain the high incorporation of glucose-derived ^13^C carbons (Fig. 1 *A* and *D*). The low isotopic enrichment of pyruvate may be due to the high abundance of nonlabeled pyruvate in the extracellular medium (Fig. 1*D*). Taken together with the relative intracellular abundance of the metabolites, the kinetic labeling data demonstrated a faster metabolic flux of succinate-derived carbons than glucose-derived carbons for the PVD biosynthetic precursors derived from the TCA cycle (Fig. 1*D*).

Previous work (9) on Fe-limited *P. putida* cells grown on glucose has revealed that, in addition to a sevenfold decrease in biomass growth and a twofold decrease in glucose consumption relative to Fe-replete cells, enhanced gluconate secretion accounted for up to 50% of consumed glucose in the Fe-limited cells. The periplasmic oxidation of glucose to gluconate could be energetically beneficial to the Fe-limited cells by generating reducing equivalents (as reduced ubiquinone [UQH_2_]) without exerting the expense of processing glucose intracellularly through a compromised metabolism (42). The metabolic overflow via gluconate secretion may explain both the slow metabolic flux of assimilated glucose toward siderophore precursors and the low siderophore yield observed here (Fig. 1 *B* and *D*). Metabolic overflow was also reported for Fe-limited *E. coli* (5), which secreted 70% of consumed glucose as acetate and lactate, and Fe-limited *C. mineralivorans* (29), which secreted over 40% of consumed glucose as gluconate. With respect to the physiological response of the succinate-grown cells to Fe limitation, we recorded a threefold decrease in biomass growth and a fourfold decrease in succinate consumption, indicating that both decreased carbon assimilation and compromised metabolism were also characteristic of succinate-grown Fe-limited cells (*SI Appendix*, Fig. S2*B*). We sought to understand how succinate metabolism still could achieve carbon metabolism to benefit siderophore biosynthesis in Fe-limited cells.

### Enhanced Anaplerosis in Fe-Deficient Cells Promotes Succinate Carbon Retention in the TCA Cycle.

**Fig. 2.**
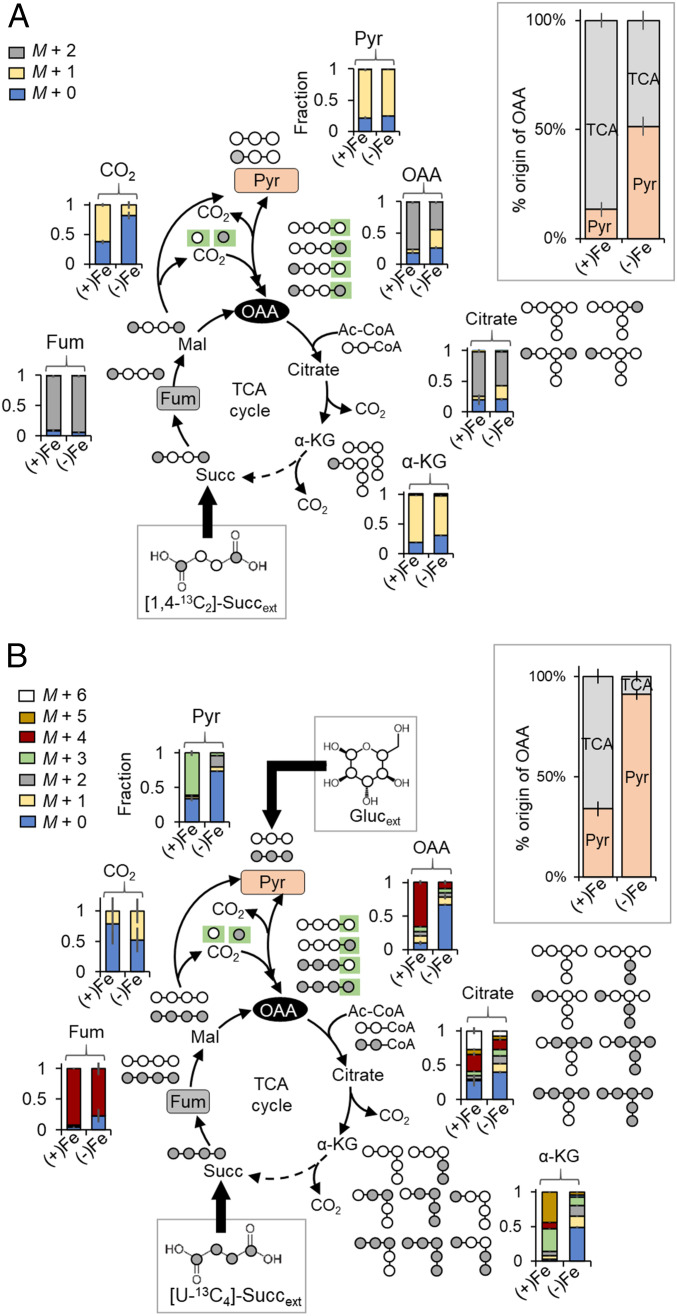
Anaplerotic flux favored over gluconeogenic flux to promote succinate carbon retention in the TCA cycle. Carbon mapping, metabolite labeling, and flux ratio analysis of (*A*) assimilated [1,4-^13^C_2_]-succinate or (*B*) assimilated [U-^13^C_4_]-succinate with unlabeled glucose in exponentially growing *P. putida* KT2440 cells cultured in Fe-replete [(+)Fe] and intermediate Fe-limited [(−)Fe] conditions. Dashed arrow represents a minor flux. Addition of CO_2_-derived carbon in OAA synthesis is shown with a green box. The measured data (mean ± SD) were from three biological replicates. The metabolite abbreviations are as described in the Fig. 1 legend.

We investigated metabolic routing in Fe-replete and Fe-limited *P. putida* KT2440 grown on [1,4-^13^C_2_]-succinate alone (Fig. 2*A*). This doubly ^13^C-labeled succinate would first generate doubly ^13^C-labeled fumarate and doubly ^13^C-labeled OAA through the TCA cycle and, after decarboxylation through gluconeogenic fluxes, singly ^13^C-labeled pyruvate; subsequent runs through the TCA cycle would yield nonlabeled metabolites (Fig. 2*A*). We measured nonlabeled (26%), singly ^13^C-labeled (29%), and doubly ^13^C-labeled (45%) fractions of OAA in Fe-limited cells, whereas Fe-replete cells only had 5% singly ^13^C-labeled fractions (Fig. 2*A*). The only metabolic route for singly ^13^C-labeled OAA would be the carboxylation of nonlabeled pyruvate by labeled CO_2_ or carboxylation of singly ^13^C-labeled pyruvate by nonlabeled CO_2_ (Fig. 2*A*). Using the labeling data and flux ratio analysis, we quantified the relative contribution of TCA cycle-derived flux versus anaplerotic flux from pyruvate to OAA biosynthesis (Fig. 2*A*). Compared to Fe-replete cells, the contribution from the TCA cycle was reduced by nearly half (from 87 to 49%) and the anaplerotic flux from pyruvate became the primary contributor to OAA biosynthesis (from 15 to 51%) in the Fe-limited cells, thus explaining the observed retention of succinate-derived carbons in PVD precursors from the TCA cycle (Figs. 1*D* and 2*A*).

**Fig. 3.**
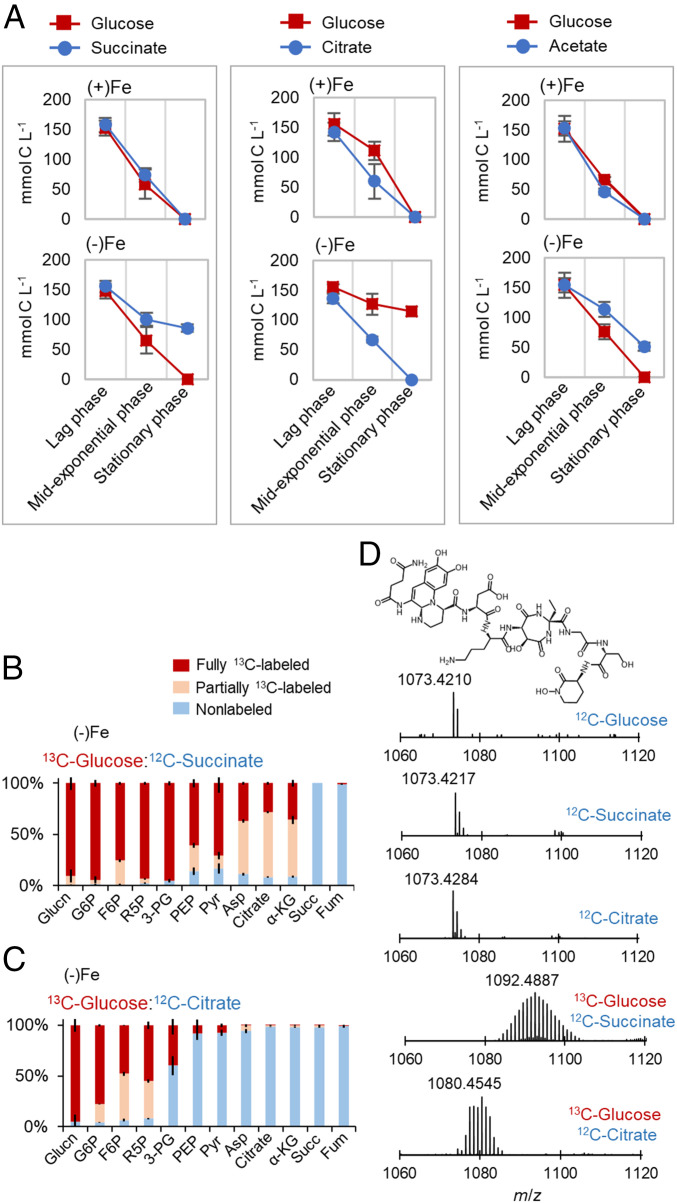
Fe-limited cells trigger substrate hierarchy during mixed-substrate usage. (*A*) Substrate consumption during growth of Fe-replete [(+)Fe] and intermediate Fe-limited [(−)Fe] *P. putida* KT2440 on carbon-equivalent mixture with (*Left*) 1:1 glucose:succinate, (*Middle*) 1:1 glucose:citrate, or (*Right*) 1:1 glucose:acetate mixture; samples were obtained during lag phase, midexponential phase, and at the onset of stationary phase. Intracellular metabolite labeling in exponentially growing *P. putida* KT2440 cells after assimilation of (*B*) [U-^13^C_6_]-glucose and unlabeled succinate or (*C*) [U-^13^C_6_]-glucose and unlabeled citrate. (*D*) Electrospray-ionized monoisotopic positive ions ([M + H]^+^) of the primary PVD siderophore secreted by *P. putida* KT2440 during intermediate Fe-limited growth on unlabeled glucose (^12^C-glucose) alone, unlabeled succinate (^12^C-succinate) alone, unlabeled citrate (^12^C-citrate) alone, fully labeled glucose with unlabeled succinate (^13^C-glucose:^12^C-succinate), or fully labeled glucose with unlabeled citrate (^13^C-glucose:^12^C-succinate). Measured data (mean ± SD) were from biological replicates (*n* = 3). The metabolite abbreviations are as described in the Fig. 1 legend.

**Fig. 4.**
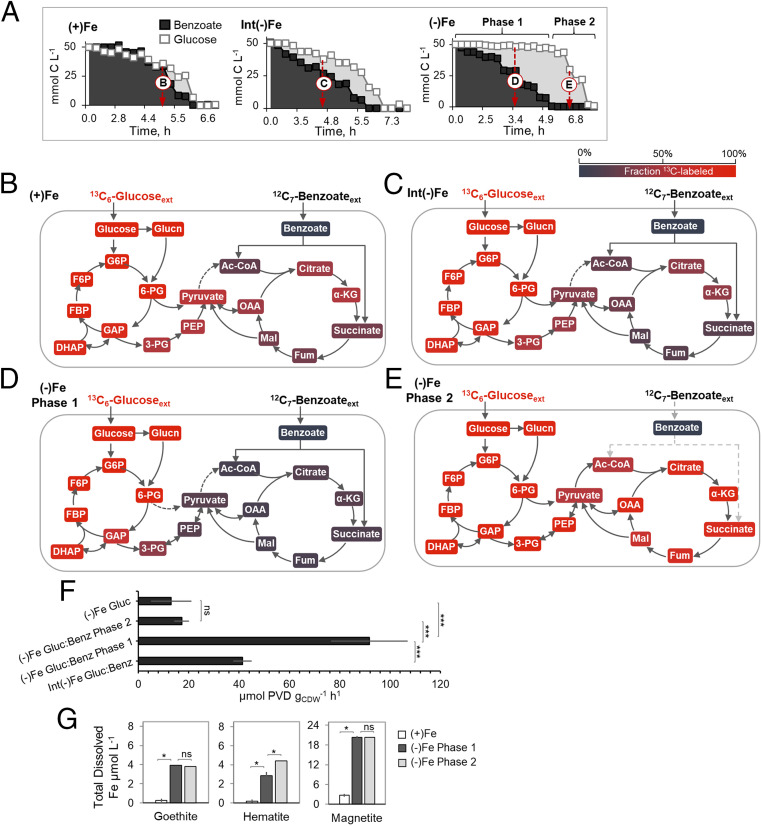
Enhanced siderophore production associated with preferential carbon usage and hierarchy in carbon metabolism. (*A*) Kinetics of substrate depletion (in % of total C) by *P. putida* KT2440 grown on 1:1 glucose:benzoate mixture under, from *Left* to *Right*, Fe-replete [(+)Fe], intermediate Fe-limited [Int(−)Fe], and Fe-limited [(−)Fe] conditions. (*B*–*E*) Intracellular metabolite labeling after assimilation of [U-^13^C_6_]-glucose and unlabeled benzoate during (*B*) (+)Fe, (*C*) Int(−)Fe, (*D*) (−)Fe phase 1, and (*E*) (−)Fe phase 2 conditions; metabolites with glucose-derived ^13^C carbons and benzoate-derived unlabeled carbons are depicted in red and dark blue shades, respectively. Sampling times for the metabolite labeling data shown in *B–E* are shown by the white arrows in *A*; detailed graphs of the metabolite labeling data are in *SI Appendix*, Fig. S3. (*F*) Siderophore production rate (µmol PVD g_CDW_^−1^ h^−1^) during (−)Fe growth on only glucose or growth on the glucose:benzoate (Gluc:Benz) mixture under int(−)Fe), (−)Fe phase 1, and phase 2 conditions (one-way ANOVA and Tukey’s studentized range test: ns, not statistically significant; ***P < 0.001). (*G*) Total dissolved Fe (µmol Fe L^−1^) from the dissolution of Fe-bearing minerals (goethite, hematite, and magnetite; 1 g L^−1^) following reactions with bacterial secretions obtained with *P. putida* KT2440 cells at the end of growth on the Gluc:Benz mixture under (+)Fe, (−)Fe phase 1, and (−)Fe phase 2 conditions (two-tailed *t* test: *P < 0.05; ns, not significant). In *A* and *B*, the (+)Fe consumption and labeling data used here as comparative reference data were obtained from previously conducted experiments (43). All data were obtained from three biological replicates. The metabolite abbreviations are as described in the Fig. 1 legend.

When the cells were fed on a mixture of [U-^13^C_4_]-succinate with unlabeled glucose, there was an increased incorporation of glucose-derived nonlabeled carbons into OAA and the oxidative side of the TCA cycle in the Fe-limited cells compared to Fe-replete cells, demonstrating again the preference of anaplerosis over gluconeogenesis in response to Fe limitation (Fig. 2*B*). Specifically, two-thirds of OAA were derived from the TCA cycle and one-third from pyruvate in Fe-replete cells, whereas, in the Fe-limited cells, only 10% of OAA was derived from the TCA cycle and 90% was from pyruvate (Fig. 2*B*). The preferential incorporation of ^13^C-glucose carbons indicated a preference for glucose utilization over succinate utilization in the Fe-limited cells (Fig. 2*B*). To shed light on Fe-dependent substrate selectivity and metabolic remodeling, we investigated different scenarios of substrate mixtures (Figs. 3 and 4).

### Iron Limitation Triggers Substrate Selectivity during Mixed-Substrate Growth.

We monitored substrate depletion during feeding on 1:1 carbon-equivalent mixture of glucose with succinate, citrate, acetate, or benzoate (Figs. 3*A* and 4*A*). There was equal or near-equal consumption of both substrates by Fe-replete cells but, in response to Fe limitation, we observed consistently that one substrate was consumed preferentially (Figs. 3*A* and 4*A*). With the Fe-limited cells, on one hand, glucose was depleted two times faster than succinate or 50% faster than acetate but, on the other hand, citrate was depleted six times faster than glucose and benzoate was depleted at least two times faster than glucose (Figs. 3*A* and 4*A*). The preferential consumption of citrate over glucose may be because citrate both served as a Fe chelator and provided carbon influx toward PVD biosynthesis (Fig. 1*A*). The role of citrate in Fe scavenging or in relieving Fe-limited growth has been reported in *P. putida* strains (44), the bacterium *Bacillus cereus* (45), the fungus *Aspergillus niger* (46), the bacterial fish pathogen *Photobacterium damselae* (47), and in the cyanobacterium *Trichodesmium erythraeum* (48). In our starting nutrient composition, we computed that 98% of the exogenous Fe was complexed with citrate, emphasizing the role of citrate as a notable Fe chelator (*SI Appendix*, Table S1).

When glucose was provided with benzoate (a gluconeogenic substrate that does not bind Fe strongly), there were near-equal consumption rates of both substrates in the Fe-replete cells (19.8 ± 4.4 mmol C g_CDW_^−1^ h^−1^ and 27.2 ± 8.1 mmol C g_CDW_^−1^ h^−1^ for glucose and benzoate, respectively; g_CDW_ is the cell dry weight in grams) as previously reported (43), but the Fe-limited cells preferably consumed benzoate over glucose, whereby decrease in Fe availability led to an increase in this carbon hierarchy (Fig. 4*A*). At the intermediate Fe-limited condition, benzoate consumption was 57% faster than glucose consumption and, for the severe Fe-limited condition, there were two clear phases of substrate depletion wherein benzoate depletion (64.5 ± 1.2 mmol C g_CDW_^−1^ h^−1^) occurred rapidly prior to the start of the glucose depletion (15.2 ± 0.2 mmol C g_CDW_^−1^ h^−1^) (Fig. 4*A*). To understand the intracellular metabolism associated with the different aforementioned substrate hierarchies, we performed ^13^C-metabolomics profiling of *P. putida* KT2440 cells grown on [U-^13^C_6_]-glucose with unlabeled succinate, unlabeled citrate, or unlabeled benzoate (Figs. 3 *B*–*D* and 4 *B*–*E*).

### Prioritizing Anaplerotic Flux versus Gluconeogenetic Flux Depends on Carbon Influx into the TCA Cycle.

In the mixture with ^13^C-glucose with unlabeled succinate, the assimilation flux of the glucose-derived ^13^C carbons in the TCA cycle was preferred over the flux of succinate-derived nonlabeled carbons, in accordance with enhanced flux from lower glycolysis (i.e., anaplerosis) toward the TCA cycle as already discussed (Figs. 2*B* and 3*B*). However, the assimilation flux of the citrate-derived nonlabeled carbons preferentially populated both the TCA cycle and lower glycolysis, implying a preference of gluconeogenic flux over anaplerotic flux when the carbon source was fed directly into the oxidative side of the TCA cycle (Fig. 3*C*). We confirmed that these different hierarchies in the carbon selectivity were reflected in the isotopic enrichment of the resulting PVD produced (Fig. 3*D*).

We compared the isotopologue distribution of the major [M + H]^+^ PVD ion in the bacterial secretions following growth on unlabeled substrate alone (glucose, succinate, or citrate) or a mixture of ^13^C-labeled glucose with unlabeled succinate or unlabeled citrate (Fig. 3*D*). The unlabeled PVD [M + H]^+^ ion (mass-to-charge ratio [*m*/*z*] of ∼1,073.4210) detected during growth on each unlabeled single substrate was replaced with variable ^13^C enrichment of this ion in agreement with the carbon selectivity (Fig. 3*D*). From the Gaussian isotopologue distribution of the PVD ion, the highest abundant peak had a *m*/*z* value of 1,092.4887 during feeding on the mixture with ^13^C-glucose and unlabeled succinate, but the corresponding peak had a *m*/*z* value of 1,080.4545 during feeding on the mixture with ^13^C-glucose and unlabeled citrate, indicating a higher incorporation of glucose-derived ^13^C carbons into the PVD structure in the presence of succinate than in the presence of citrate (Fig. 3*D*). Although the initial catabolism of succinate and citrate both require Fe-containing enzymes (respectively, succinate dehydrogenase and aconitate hydratase), our data implied that the assimilation of these two substrates was under different metabolic regulation.

With respect to the glucose:benzoate conditions, the extent of carbon hierarchy was enhanced upon worsening Fe deficiency (Fig. 4 *A*–*E*). In Fe-replete cells, the catabolism of glucose- and benzoate-derived carbons was nonuniform throughout the metabolic network, as detailed in a previous study (43) (Fig. 4*B*). The metabolites in initial glucose catabolism (gluconate, glucose-6-phosphate [G6P]), the ED pathway (6-PG, dihydroxyacetone-phosphate [DHAP]), the upper Embden–Meyerhof–Parnas (EMP) pathway (G6P, fructose-6-phosphate [F6P], fructose-1,6-bisphosphate [FBP]), and the PP pathway (xylulose-5-phosphate [Xu5P], ribose-5-phosphate [R5P], and sedoheptulose-7-phosphate [S7P]) were populated primarily by glucose-derived ^13^C-labeled carbons (by over 95%) (Fig. 4*B*). The metabolites in the TCA cycle (OAA, citrate, α-KG, fumarate, and malate) were derived from both nonlabeled benzoate carbons (60% or higher) and glucose ^13^C carbons (about 40% or lower) (Fig. 4*B* and *SI Appendix*, Fig. S3). The metabolites upstream of the TCA cycle (3-PG, PEP, and pyruvate) were enriched with glucose-derived ^13^C-carbons with some minor fractions of benzoate-derived nonlabeled carbons (Fig. 4*B* and *SI Appendix*, Fig. S3). With the intermediate Fe deficiency, the fraction of benzoate-derived nonlabeled carbons was increased in metabolites both upstream of and throughout the TCA cycle (Fig. 4 *C* and *D* and *SI Appendix*, Fig. S3). At the most severe Fe-limited condition, the first phase of substrate utilization led to preferential incorporation of nonlabeled benzoate carbons over ^13^C-glucose carbons, including an appreciable fraction (up to 25%) of partially ^13^C-labeled fraction of metabolites in initial glucose catabolism (G6P, F6P, and FBP) (Fig. 4*D* and *SI Appendix*, Fig. S3). In the second phase, the switch to glucose after benzoate depletion led to high ^13^C-labeled enrichment (over 70%) of all metabolites in the TCA cycle (Fig. 4 *A* and *E* and *SI Appendix*, Fig. S3). In sum, the metabolite labeling patterns from the different mixture scenarios (glucose with succinate, glucose with citrate, or glucose with benzoate) revealed that Fe deficiency led to a rerouted carbon metabolism in association with a hierarchy for gluconeogenic substrates with direct influx into the oxidative side of the TCA cycle over the anaplerotic flux from glucose-derived carbons (Figs. 3 *B* and *C* and 4 *B*–*E*).

### Carbon Hierarchy Leads to Enhanced Siderophore Production and Fe Scavenging.

We evaluated explicitly the consequence of the carbon hierarchy of benzoate over glucose on siderophore secretion and Fe scavenging (Fig. 4 *F* and *G*). Compared to the PVD secretion rate under the intermediate Fe-limited condition (41.4 ± 3.59 µmol g_CDW_^−1^ h^−1^), the lowest Fe availability led to a near 2-fold increase in the PVD secretion rate (91.8 ± 15.2 µmol g_CDW_^−1^ h^−1^) during the first phase when benzoate was the primary substrate consumed from the mixture (Fig. 4 *A* and *F*). During the second phase when growth was supported only by glucose from the mixture, there was a 5-fold decrease in the PVD secretion rate (17.5 ± 2.86 µmol g_CDW_^−1^ h^−1^) relative to the first phase; Fe-limited cells grown on glucose alone had a similar PVD secretion rate (13.0 ± 8.0 µmol g_CDW_^−1^ h^−1^) (Fig. 4*F*). Given reported overflow of oxidized glucose as secreted gluconate (9), we compared the gluconate secretion rates during the two phases of substrate consumption in the Fe-limited cells and found a 200-fold increase in gluconate secretion (from 11.6 ± 4.6 µmol g_CDW_^−1^ h^−1^ to 2.3 ± 0.3 mmol g_CDW_^−1^ h^−1^) during the glucose-consumption phase compared to the benzoate-consumption phase (*SI Appendix*, Fig. S4*A*). Therefore, the preferential uptake of benzoate-derived carbons resulted in the highest rate of PVD production and bypassed the excess carbon loss via gluconate secretion (Fig. 4 *A*–*F* and *SI Appendix*, Figs. S4 *A* and *B* and S5).

Moreover, we found that the PVD-containing bacterial secretions from the glucose:benzoate growth condition led to significant levels of dissolved Fe, ranging from 2.8 to 20.3 µM of Fe on average, extracted from the aforementioned Fe-oxide minerals (Fig. 4*G*). Across the two consumption phases, the total extent of Fe scavenged from the minerals was largely achieved by the bacterial secretions following the benzoate-consumption phase; following the glucose-consumption phase, the dissolved Fe concentration either remained the same (for goethite and magnetite) or was increased by only 35% (for hematite) (Fig. 4*G*). Thus, the enhanced PVD secretion associated with the carbon hierarchy optimized Fe scavenging.

### Enzymatic Regulation Versus Metabolic Regulation of Fe-Dependent Carbon Hierarchy to Enhance Siderophore Production.

**Fig. 5.**
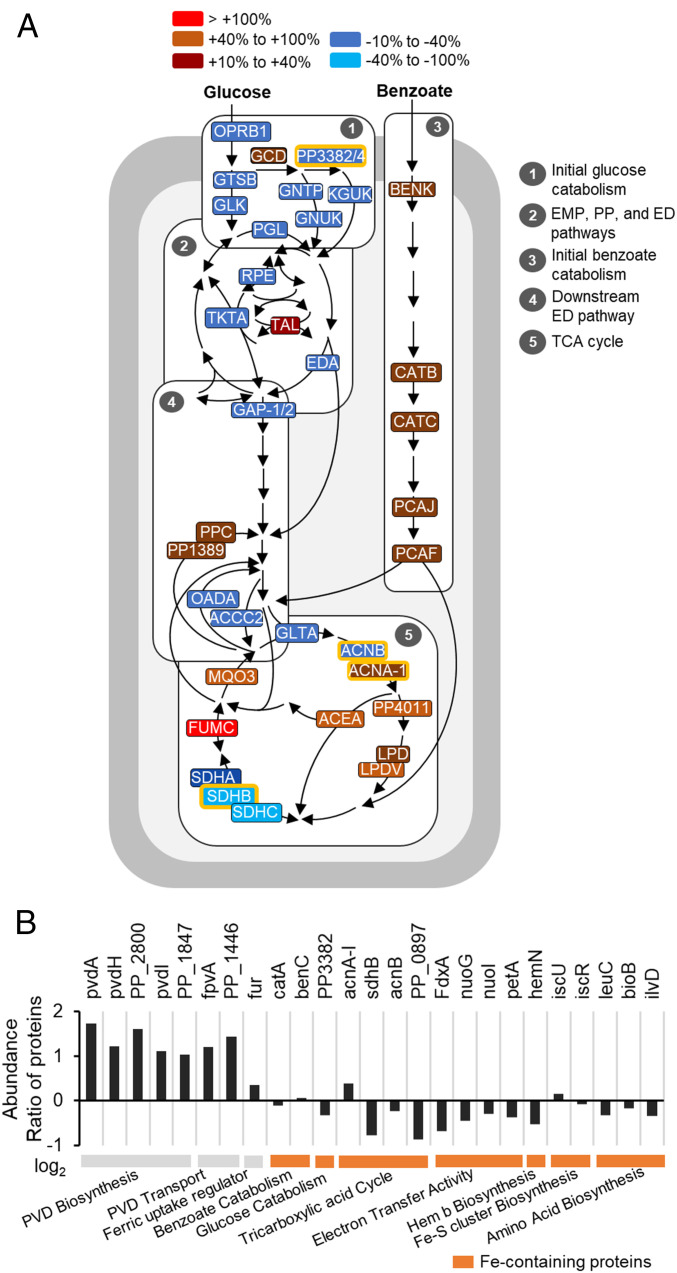
Fe-dependent carbon selectivity and siderophore biosynthesis facilitated by protein abundance changes. (*A*) Percent changes (greater than or less than 10%) in proteins involved in the central carbon metabolism of *P. putida* KT2440 cells grown on the glucose:benzoate mixture under Fe-limitation versus Fe-replete conditions. (*B*) Fold change (expressed in log2 of ratio of Fe-limitation data versus Fe-replete data) in the abundance of proteins involved in PVD biosynthesis and transport, proteins involved in Fe regulation, and Fe-containing metabolic proteins. The Fe-limited data were obtained during the first phase of Fe-limited substrate consumption depicted in Fig. 4*A*. The total profiling of protein abundance ratios and data statistics is in *SI Appendix*, Table S2. Data were obtained from three biological replicates.

**Fig. 6.**
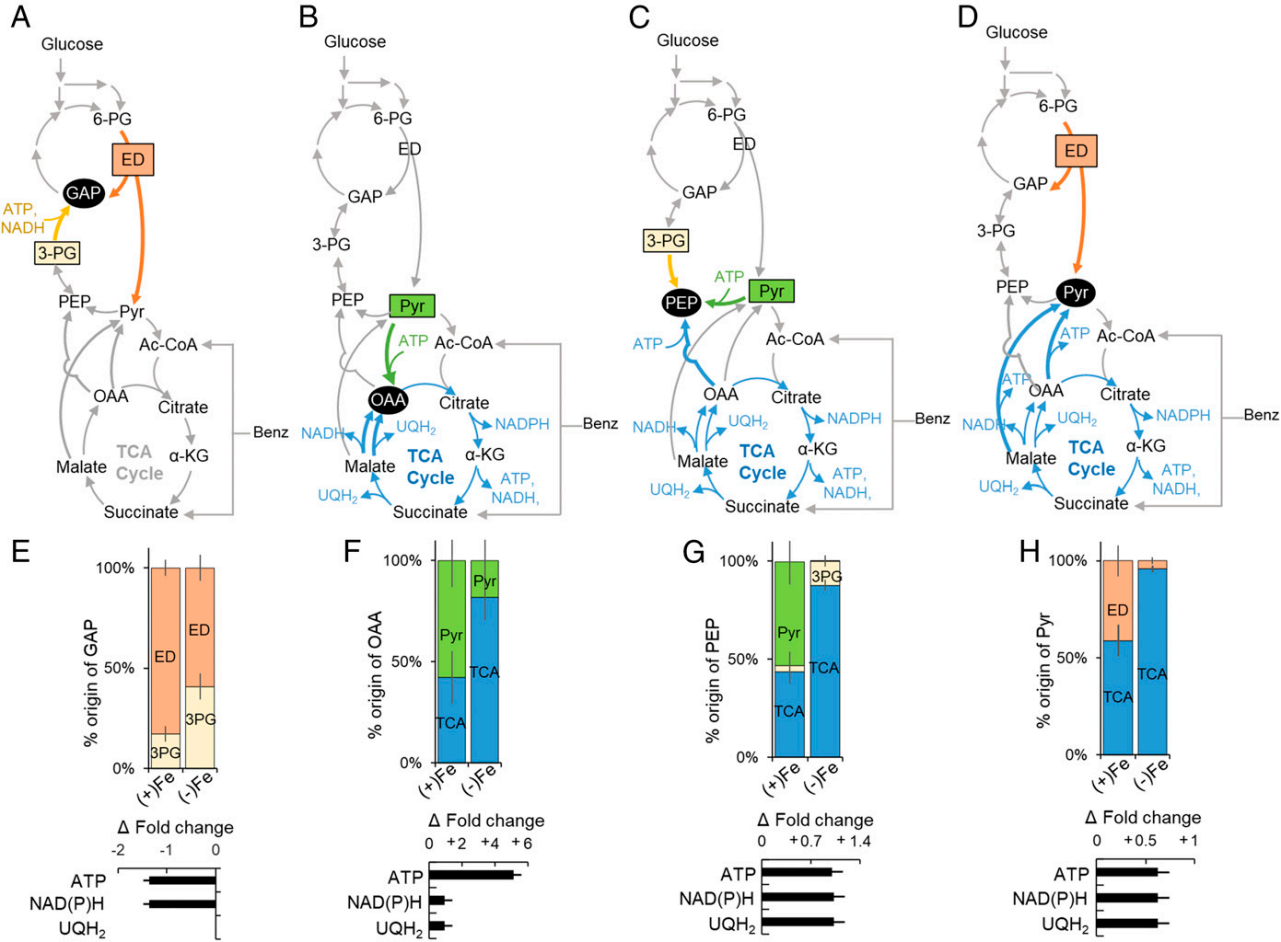
Metabolic remodeling of carbon fluxes in response to Fe deficiency. (*A*–*D*) Scheme of the metabolic routes for the biosynthesis of four metabolic nodes: (*A*) GAP derived from 3-PG or the ED pathway, (*B*) OAA derived from pyruvate or malate via the TCA cycle, (*C*) PEP derived from 3-PG, Pyr, or OAA via the TCA cycle, and (*D*) Pyr derived from the ED pathway or from the TCA cycle (via OAA and malate). (*E*–*H*) (*Top*) Fe-dependent percentage (mean ± SD) of each precursor compound or pathway for each metabolic node and (*Bottom*) estimated fold-change increase or decrease (mean ± SD) in ATP, UQH_2_, and NAD(P)H in Fe-limited cells relative to Fe-replete cells of *P. putida* KT2440 during growth on the glucose:benzoate mixture. The Fe-limited cell data were obtained during the first phase of Fe-limited substrate consumption depicted in Fig. 4*A*. Analyses were conducted using metabolite labeling data from three biological replicates (*SI Appendix*, Fig. S3). The metabolite abbreviations are as described in the Fig. 1 legend.

We explored how changes in enzyme abundance versus changes in metabolic fluxes facilitated the aforementioned substrate hierarchy to enhance PVD production (Figs. 5 and 6). We compared data obtained with Fe-limited cells during the first (or benzoate-only) consumption phase from the glucose:benzoate mixture to those obtained with Fe-replete cells grown on the same carbon mixture (Figs. 5 and 6). Here we highlight changes in protein abundance that were 10% less or greater in the Fe-limited cells than in the Fe-replete cells (Fig. 5*A* and *SI Appendix*, Table S2). Along with the proteomics analysis, we used the ^13^C-metabolomics data illustrated in Fig. 4 *B* and *C*, to perform flux ratio analyses and characterize changes in the biosynthetic routes at four key metabolic nodes: OAA, GAP, PEP, and pyruvate (Fig. 6 *A*–*D*).

Our proteomics profiling quantified a total of 52 proteins involved in initial substrate catabolism and central carbon metabolism (Fig. 5*A*). Consistent with the decreased glucose uptake, 9 of the 10 identified enzymes involved in initial glucose catabolism were depleted (by up to −38%) in Fe-limited cells relative to the Fe-replete cells (Figs. 4*A* and 5*A*). However, glucose dehydrogenase (gcd) was elevated by 17%, in accordance with reported increase in gluconate secretion (9) in Fe-limited cells fed on glucose (Fig. 5*A* and *SI Appendix*, Fig. S4*A*). With respect to initial benzoate catabolism, five enzymes (benK, catB, catC, pcaJ, and pcaF) were elevated (by up to 20%) in the Fe-limited cells (Fig. 5*A*). Of the 7 enzymes quantified in the upper EMP, PP, and ED pathways, the levels of 4 were depleted (by 30%) in the Fe-limited cells (Fig. 5*A*). Notably, in accordance with the depletion (by 29%) in the aldolase enzyme (eda) that splits 6-PG into GAP and pyruvate in the ED pathway, the flux analysis determined a decreased contribution of the ED pathway in GAP biosynthesis in the Fe-limited cells (59%) compared to the Fe-replete cells (83%) (Figs. 5*A* and 6 *A* and *E*). Thus, the carbon selectivity of benzoate over glucose in the Fe-deficient cells was facilitated by changes in the abundance of metabolic enzymes involved in transport and initial catabolism of each substrate (Fig. 5*A*).

For OAA biosynthesis, the metabolic flux analysis determined a near-equal investment of pyruvate (58%) and the TCA cycle via malate (42%) in the Fe-replete cells, whereas there was a significant dependency on the TCA cycle (82%) with a small contribution from pyruvate (18%) in the Fe-limited cells (Fig. 6 *B* and *F*). In agreement with these flux distributions, the level of malate:quinone oxidoreductase (mqo3) for the conversion of malate to OAA was increased (by 62%) and the level of pyruvate carboxylase (accC2) for the carboxylation of pyruvate to OAA was decreased (by 21%) in the Fe-limited cells compared to the Fe-replete cells (Fig. 5*A*). An increased contribution of gluconeogenic reactions from the TCA cycle at the expense of anaplerosis was also evident in the flux ratio analyses for PEP and pyruvate biosynthesis (Fig. 6 *G* and *H*).

Compared to the contributions from the TCA cycle (43%) and pyruvate (53%) for PEP biosynthesis in the Fe-replete cells, the majority of PEP was synthesized from the TCA cycle via OAA (88%) in the Fe-limited cells (Fig. 6*C*). Of the 12 quantified enzymes in the gluconeogenic/anaplerotic junction pathways, the 2 enzymes involved in the gluconeogenic synthesis of PEP via the OAA node of the TCA cycle were elevated in the Fe-limited cells compared to the Fe-replete cells: phosphoenolpyruvate carboxylase (ppc, by 25%) and oxaloacetate decarboxylase (PP1389, by 19%) (Fig. 5*A*). For pyruvate biosynthesis, pyruvate was made from both the ED pathway (41%) and the TCA cycle (59%) in Fe-replete cells but only 4% of pyruvate was derived from the ED pathway and the major biosynthetic source was the TCA cycle (96%) in the Fe-limited cells (Fig. 6*H*). Incongruent with the metabolic flux change in pyruvate biosynthesis, the level of oxaloacetate decarboxylase (oadA) involved in the decarboxylation of OAA to pyruvate was decreased (by 32%) in the Fe-limited cells compared to the Fe-replete cells (Fig. 5*A*).

Within the TCA cycle, the abundances of 6 metabolic enzymes (mqo3, acnA-1, PP4011, fumC, lpd, and lpdV) were elevated in the Fe-limited cells (Fig. 5*A*). Most significant was a threefold increase in fumC, which converts fumarate to malate (Fig. 5*A*). The levels of the enzymes involved in succinate synthesis were also elevated: lpd (by 56%), lpdV (by 22%), and pcaF (by 14%) (Fig. 5*A*). Notably, the 2 Fe-containing enzymes in the TCA cycle were depleted: aconitate hydratase (acnB, by −15%) and succinate dehydrogenase (sdhB, by −42% decrease) (Fig. 5 *A* and *B*). Additionally, in the Fe-limited cells, there was an increased abundance of isocitrate lyase (acea, by 10%) in the glyoxylate shunt, which was consistent with reported induction of the glyoxylate shunt from elevated acetyl-CoA that would be expected from benzoate catabolism (49). Furthermore, from the global proteomics profiling, we found that the levels of 17 Fe-containing enzymes either remained unchanged or were depleted (by up to −82%) in the Fe-limited cells relative to the Fe-replete cells (Fig. 5*B*). Toward promoting Fe acquisition, proteins involved in PVD biosynthesis and transport were elevated by over 200% in Fe-limited cells, in agreement with reported up-regulation of genes associated with PVD in Fe-limited *Pseudomonas* species (6) (Fig. 5*B*).

Using the metabolic flux ratio analyses, we estimated the changes in the energetic yields afforded to the Fe-limited cells due to the metabolic rerouting relative to the Fe-replete cells (Fig. 6 *E*–*H*). The increased gluconeogenic flux from the TCA cycle in the biosynthesis of OAA and pyruvate in Fe-limited cells led to a greater than fivefold increase in adenosine triphosphate (ATP) yield and a nearly 150% increase each in reduced nicotinamide adenine (diphosphate) [NAD(P)H] and UQH_2_ (Fig. 6 *F* and *H*). However, the change in the biosynthetic route for GAP was energetically unfavorable but, since the net consumption of ATP and NAD(P)H from 3-PG to GAP was less than their net generation from the biosynthesis of OAA and pyruvate (43), the overall remodeled metabolic network would still lead to favorable energy fluxes in the Fe-limited cells (Fig. 6*E*).

### Carbon Flux Demand for Siderophore Biosynthesis Drives Metabolic Flux Rerouting.

**Fig. 7.**
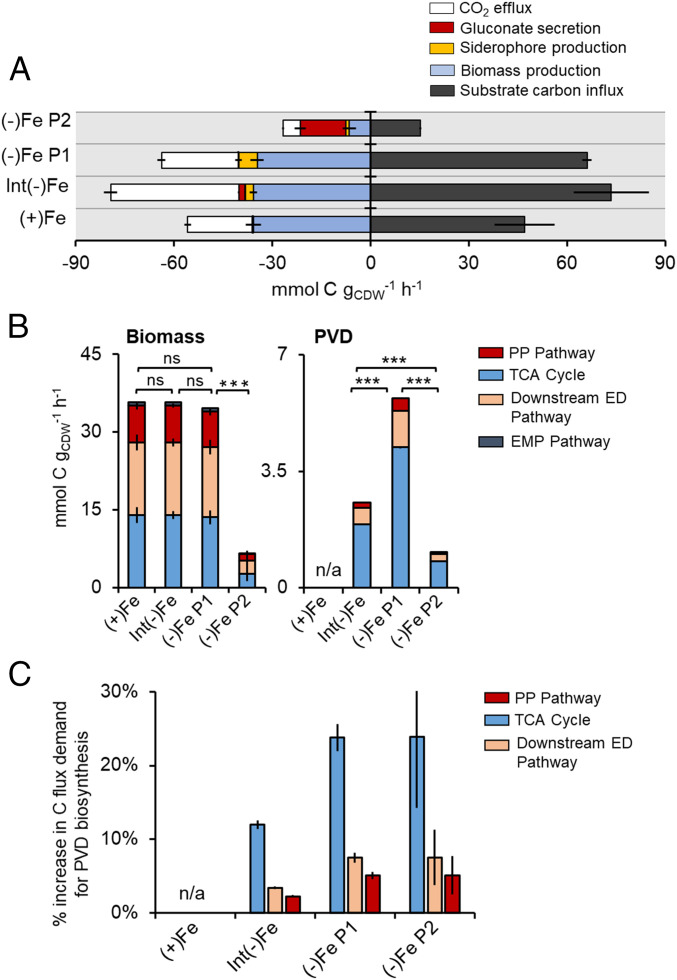
Metabolic pathway flux demand for biomass growth versus siderophore biosynthesis. (*A*) Measured carbon influx, measured effluxes (gluconate secretion, siderophore production, and biomass production), and estimated CO_2_ production (mmol C g_CDW_ h^−1^) in *P. putida* KT2440 grown on 1:1 glucose:benzoate mixture under Fe-replete [(+)Fe], intermediary Fe-limited [Int(−)Fe], Fe-limited [(−)Fe] phase 1 (P1), and (−)Fe phase 2 (P2) conditions. Color code: CO_2_ production (green), gluconate secretion (yellow), siderophore production (orange), biomass production (blue), and substrate carbon influx (gray). (*B*) Calculated pathways flux demand (mmol C g_CDW_^−1^ h^−1^) required from central carbon metabolism to sustain (*Left*) measured biomass growth and (*Right*) biosynthesis of measured PVD secretion rate (one-way ANOVA, Tukey’s studentized range test: ns, not statistically significant; ***P < 0.001). (*C*) Percentage (%) increase in metabolic flux demand for PVD biosynthesis relative to flux demand for biomass growth alone. Color code for *B* and *C*: EMP pathway, dark blue; PP pathway, red; downstream ED pathway, light orange; and TCA cycle, light blue. All data, shown as mean ± SD, were obtained from three biological replicates.

Thus far, our data have demonstrated that hierarchy in carbon metabolism promoted fluxes in the TCA cycle and led to enhanced PVD production, despite the depletion of Fe-containing and other proteins in the TCA cycle and connected metabolic pathways (Fig. 4*F*). We conducted a quantitative analysis of the metabolic flux demands for biomass growth versus siderophore biosynthesis during *P. putida* KT2440 growth on the glucose:benzoate mixture across the different Fe conditions. We combined measured data (growth rates, carbon consumption rates, siderophore production rates, and metabolite secretion rates) and computed data (biomass biosynthetic demand, PVD biosynthetic demand, and CO_2_ efflux) (Fig. 7*A*). The metabolic flux demand to sustain biomass growth was based on genome-scale cellular stoichiometry (50) and the PVD biosynthetic demand was based on the pre-PVD structure of the primary PVD secreted by *P. putida* KT2440 (10, 40) (Fig. 7*A*). Guided by previous metabolic flux analyses (9, 43), the CO_2_ efflux was estimated by performing flux balance analysis using the ^13^C-labeling patterns obtained at the different Fe conditions (*SI Appendix*, Table S3 and Fig. S2 and Fig. 7*A*).

Compared to the carbon influx rate in the Fe-replete cells (47.0 ± 9.2 mM C g_CDW_^−1^ h^−1^), the corresponding rate was 56% higher in the intermediate Fe-limited condition (73.5 ± 11 mM C g_CDW_^−1^ h^−1^), 41% higher in first phase of the severe Fe-limited condition (66.1 ± 1.4 mM C g_CDW_^−1^ h^−1^), and threefold lower in the second phase (Fig. 7*A*). Siderophore secretion rate accounted for only 3.5%, 8.6%, and 7.1% of the carbon influx rate for the intermediate Fe-limited condition, first phase, and second phase of the severe Fe-limited condition, respectively (Fig. 7*A*). The primary carbon efflux was instead for biomass production (about 50% or higher) in all conditions, except for the latter condition (i.e., second phase of the Fe-limited condition) wherein 52% of the carbon efflux was due to gluconate secretion (Fig. 7*A*). Compared to the CO_2_ efflux estimated for the Fe-replete cells (19.6 ± 0.98 mM C g_CDW_^−1^ h^−1^) (43), this value was twofold higher in the intermediate Fe-limited condition (39.1 ± 2.1 mM C g_CDW_^−1^ h^−1^), 20% higher in the first phase of severe Fe-limited condition (23.4 ± 1.2 mM C g_CDW_^−1^ h^−1^), and 73% lower in the second phase of severe Fe-limited condition (5.24 ± 0.3 mM C g_CDW_^−1^ h^−1^) (Fig. 7*A*). These values implied that siderophore biosynthesis involved CO_2_-generating metabolic reactions. For the second phase of the severe Fe-limited condition, there was a large discrepancy between the total carbon efflux (26.7 ± 2.7 mM C g_CDW_^−1^ h^−1^) and the carbon influx (15.2 ± 0.2 mM C g_CDW_^−1^ h^−1^), which we attributed to the unaccounted contribution of carbon retention from the first phase beyond the measured carbon influx in the second phase (Fig. 7*A*). We also considered the possible secretion of other organic acid metabolites but their summed concentration was negligible (<1 mM C g_CDW_^−1^ h^−1^) across all conditions (*SI Appendix*, Table S4).

Metabolic flux requirements to support biomass biosynthesis were satisfied primarily by the TCA cycle (∼40%), downstream of the ED pathway (∼40%) and the PP pathway (∼20%); contribution of the EMP pathway was minimal (Fig. 7*B*). Despite the relatively low fractional amount of the PVD biosynthetic efflux within the total carbon efflux, the relative metabolic flux requirement for PVD biosynthesis was significant. Specifically, due to the metabolic requirements for the PVD siderophore biosynthesis in the Fe-limited cells (Fig. 1*B*), the metabolic flux demand was increased by up to 30% from the TCA cycle and less than 10% from either the downstream ED pathway or from PP pathway (Fig. 7*C*). Therefore, the substrate hierarchy and metabolic remodeling in Fe-limited cells determined from our metabolomics data were aimed at satisfying the excess carbon flux demand required from the TCA cycle to sustain PVD biosynthesis (Figs. 5*A*, 6 *E*–*H*, and 7*C*).

## Discussion

*Pseudomonas* species are ubiquitous in environmental and physiological settings where competition for Fe acquisition is essential. Siderophore-mediated dissolution of Fe-bearing minerals in the soil matrix promotes Fe availability to plant beneficial *Pseudomonas* species (9, 51) such as *P. putida* and *P. protegens*. Siderophore is essential for pathogenic *P. aeruginosa* to be virulent and survive in the host and pathogenic *P. syringae* to compete within its microbial community (18, 20). Prior studies (6, 19, 41) stressed the gene-level regulation of Fe transport and siderophore biosynthesis in *Pseudomonas* species, but the underlying cellular carbon metabolism had remained unknown. Through multiple ^13^C-metabolomics experiments coupled with proteomics profiling, we obtained insights into the metabolic strategies that favor siderophore biosynthesis in Fe-limited *Pseudomonas* species.

**Fig. 8.**
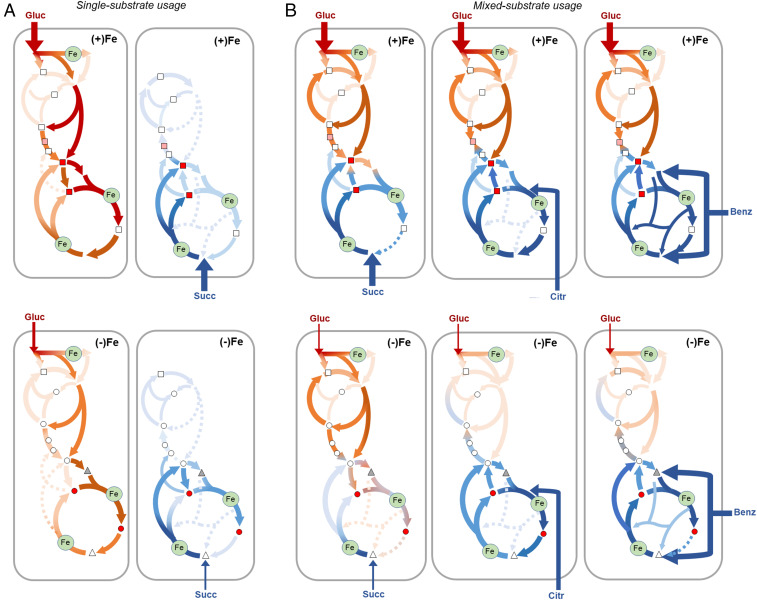
Overview of hierarchical carbon metabolism in *Pseudomonas* sp. triggered by Fe deficiency. (*A*) For singe-substrate usage, metabolomics data reveal a decreased flux toward the TCA cycle in glucose-grown cells and enhanced anaplerosis in succinate-grown cells in response to Fe limitation, resulting in an increased siderophore production in succinate-grown cells. (*B*) For mixed-substrate usage, metabolomics data capture a selectivity in carbon uptake accompanied by hierarchical carbon metabolism to favor fluxes through the TCA cycle and toward oxaloacetate and α-ketoglutarate, the two major nodes for biomass and siderophore biosynthesis. In sum, rerouted metabolic fluxes overcome decrease in Fe-containing proteins (denoted by green-filled circles) determined from the proteomics profiling, while still meeting the carbon and energy flux demands for siderophore biosynthesis. Red/orange and blue/purple shaded arrows indicate the metabolic fluxes of carbons derived, respectively, from glucose (Gluc) and a gluconeogenic substrate (succinate [Succ], citrate [Citr], or benzoate [Benz]); lighter and darker shades represent, respectively, large and small fluxes from the corresponding substrate. Color coded in accordance with their relative contribution to biomass fluxes or the PVD biosynthetic fluxes, the metabolic nodes that are precursors to biomass biosynthesis (rectangle), PVD structure (triangle), or both biomass and PVD (circle) are shown as white (0 to 10%), pink (10 to 25%), and red (above 25%) circles; the gray triangle denotes a metabolite (acetyl-CoA) used for the acyl chain present in the pre-PVD structure but not in the final PVD structure.

Despite the depletion of Fe-containing and several other enzymes both within the TCA cycle and at the gluconeogenesis–anaplerosis nodes, we shed light on how Fe-deficient cells sustained carbon and energy fluxes for PVD biosynthesis through a remodeled metabolic network: 1) a rerouted carbon metabolism in association with a substrate hierarchy led to favorable PVD yield whereby anaplerotic carbon recycling of gluconeogenic substrates promoted the siderophore yield during single-substrate usage; 2) there was a hierarchy for gluconeogenic substrates with direct influx to the oxidative side of the TCA cycle over glucose during mixed-substrate usage; and 3) in the presence of this hierarchy, there was a preference for the gluconeogenetic flux over the anaplerotic flux (Fig. 8).

Several lines of inquiries remain to be investigated. *Pseudomonas* and other bacterial species are known to exhibit carbon catabolite repression (CCR), which facilitates the utilization of preferred carbon sources from substrate mixtures. The preferential catabolism of citrate or benzoate over carbohydrates in the Fe-limited *P. putida* cells was both favorable for siderophore biosynthesis and in agreement with reported CCR of carbohydrate catabolism in favor of catabolism of organic acids (52). However, the preference for glucose over acetate or succinate in the Fe-limited *P. putida* cells was not consistent with expected CCR phenotype, implying that Fe-limited cells may exhibit different CCR regulation. Moreover, xenosiderophore uptake (53) in bacterial communities, whereby some species make use of siderophores produced by others, would decrease the metabolic burden for each species to sustain its own siderophore biosynthesis (54–57). It remains to be determined how carbon hierarchy would be distributed within a bacterial community where siderophore piracy is possible (58, 59). Interestingly, production of organic acids (such as succinate, malate, and citrate) by plant roots is reportedly increased in response to Fe deficiency (60–62), which would lead to an alteration in substrate availability to the rhizospheric bacteria (62), including *Pseudomonas* species. In light of our findings, the organic acids would present ideal substrates for recruiting Fe-deficient soil *Pseudomonas* species to promote siderophore yield and Fe scavenging. Genomics-based analysis combined with in vitro assays recently revealed that the production of growth-inhibiting versus growth-promoting siderophores determined the extent of suppression versus survival of plant pathogens in a rhizosphere microbiome (63). It remains to be investigated how the carbon dynamics within this microbiome may mediate the secretion of different siderophore types and restructure this ecological niche.

We posit that the Fe-dependent metabolic reprogramming elucidated here may be manifested widely in the environment. Recent transcriptomics and proteomics analyses uncovered an increase in carbon storage pathways in Fe-deficient *C. mineralivorans* isolated from nutrient-poor soils (29), indicating a change in carbon metabolism in response to Fe deficiency. Adaptation of a *Synechococcus* sp., a cyanobacterium, to Fe-deficient growth was shown to result in different transcript-level responses of carbon metabolism (14). Transcriptomics analysis of the marine bacterium *A. macleodi* grown under Fe limitation (58) revealed decreased dependence on Fe-containing proteins to direct cellular resources toward Fe acquisition. To shed light on these recent genomics- and transcriptomics-level data, metabolomics studies are warranted to investigate explicitly the metabolic responses to Fe deficiency with respect to both selective carbon metabolism and metabolic flux remodeling in bacteria of different genera. Beyond Fe limitation, we hypothesize that hierarchical carbon metabolism may be a critical feature in the survival strategy employed by bacteria and other microorganisms in nutrient-deficient environments. Such metabolic feature, if confirmed, would have important implications for understanding the role of metal nutrient availability in controlling carbon utilization in human and plant infections, metabolic diseases, and biogeochemical cycling in soils and oceans.

## Methods

Liquid cultures were prepared with *P. putida* KT2440 (ATCC 47054), *P. putida* S12 (ATCC 700801), and *P. protegens* Pf-5 (ATCC BAA-477) obtained from the American Type Culture Collection (ATCC). We performed metabolomics and proteomics analyses from cells grown under varying carbon sources and Fe conditions. Intracellular metabolite quantitation, metabolite labeling, and siderophore characterization were obtained using high-resolution liquid chromatography-mass spectrometry (LC-MS). Quantification in proteomics experiments was conducted using mass spectrometry-based ion trap technology. Detailed methods are provided in *SI Appendix*.

## Supplementary Material

Supplementary File

## Data Availability

Proteomics MS data and metabolomics LC-MS data are freely available in Proteome Xchange and MetaboLights depositories under identifiers PXD013605 and MTBLS1715, respectively.
