# Supplementary material for: Hierarchical routing in carbon metabolism favors iron-scavenging strategy in iron-deficient soil Pseudomonas species
Source: Proc Natl Acad Sci U S A. 2020 Dec 3;117(51):32358–69. doi: 10.1073/pnas.2016380117 (PMC7768705; doi:10.1073/pnas.2016380117)
Supplement: Supplementary File [file pnas.2016380117.sapp.pdf]

SUPPLEMENTARY INFORMATION (SI) APPENDIX

Hierarchical Routing in Carbon Metabolism Favors Iron-Scavenging Strategy in Iron-Deficient Soil *Pseudomonas* species

Caroll M. Mendonca[a,b], Sho Yoshitake[a], Hua Wei[a], Anne Werner[a], Samantha S. Sasnow[a], Theodore W. Thannhauser[c], and Ludmilla Aristilde[a,b,1]

[a]Department of Biological and Environmental Engineering, College of Agriculture and Life Sciences, Cornell University, Riley-Robb Hall, Ithaca, NY 14853; [b]Department of Civil and Environmental Engineering, McCormick School of Engineering and Applied Science, Northwestern University, Evanston, IL 60208, USA; [c]United States Department of Agriculture—Agricultural Research Service, Ithaca, NY 14853, USA.

Corresponding author information:

[1] Ludmilla Aristilde, Department of Civil and Environmental Engineering, Tech A316, Northwestern University, Evanston, IL 60208, Phone: 847-491-2999, Email: ludmilla.aristilde@northwestern.edu

This PDF file includes:

Detailed Methods, including Table S1 (pages 2-10)

Table S2 (pages 11-12)

Estimation of CO_2 Production, including Table S3 (page 13)

Table S4 (page 14)

Figure S1 to Figure S5 (pages 15-19)

SI References (pages 20-21)

Detailed Methods.

Culturing and nutrient conditions. Freeze-dried cells of *P. putida* KT2440, *P. putida* S12, and *P. protegens* Pf-5 from American Type Culture Collection (ATCC, Manassas, VA) were first plated on agar-solidified Luria-Bertani (LB) medium and stored at 4°C until use. Liquid cell cultures, which were prepared using individual colonies selected from the plates and resuspended in LB medium in 20-mL glass tubes and 125-mL or 250-mL baffled flasks inside of a G24 incubator shaker (New Brunswick Scientific, Edison, NJ) at 220 rpm and 30°C[1]. The volume of the suspended cells did not exceed one-fifth of the culturing vessels, which were covered with sponge caps to enable gas exchange and prevent contamination. The growth medium, which was pH- adjusted (pH 7.0) and filter-sterilized (0.22 µm nylon; Waters Corporation, MA, United States), contained the following major salts: 37 mM NH_4Cl, 0.80 mM $MgSO_4$, 0.034 mM $CaCl_2$. $2H_2O$, 20 mM K_2HPO_4 and 5.0 mM NaH_2PO_4. The trace metal concentrations were as follows: 0.97 µM H_3BO_3, 13 µM $CuSO_4.5H_2O$, 70 µM $ZnSO_4.5H_2O$, 5.7 µM $MnSO_4.H_2O$, 0.21 µM $NiCl_2.6H_2O$, and 1.2 µM $Na_2MoO_4.2H_2O$. We investigated different nutrient conditions by varying the concentration of both solution Fe and the types of carbon substrates. Disodium ethylene-diamine-tetra-acetic-acid (EDTA) was added as a Fe chelator and the EDTA concentration to be used was determined using the software Visual MINTEQ (version 3.1) to simulate metal speciation in the given growth medium composition, including carbon substrate concentrations as detailed below[2]. Prior to EDTA addition, the total dissolved Fe concentrations were determined by inductively coupled plasma atomic emission spectroscopy (ICP-AES, Spectro Analytical). The cells were grown under Fe-replete condition with 30 µM total dissolved unchelated Fe, intermediate Fe-limited condition with 650 nM total dissolved unchelated Fe, or Fe-limited with 38 nM total dissolved unchelated Fe—unchelated here means unchelated by EDTA (See detailed composition of the Fe speciation in Table S1). Total carbon-equivalent substrate concentrations (100 mM C or 300 mM C total) were provided as single-substrate or mixed-substrate scenarios. For the intermediate Fe-limited growth condition, we conducted experiments with glucose alone, succinate alone, citrate alone, glucose with succinate, and glucose with citrate, glucose with acetate, and glucose with benzoate; for the severe Fe-limitation, we performed experiments with glucose alone, fructose alone, succinate alone, malate alone, and glucose with benzoate. For all the substrate scenarios, we conducted Fe-replete growth experiments as reference to the intermediate and severe Fe-limited growth experiments. For the single-substrate scenarios, we used glucose (16.67 mM or 50 mM), fructose (16.67 mM), succinate (25 mM or 75 mM), malate (25 mM), or citrate (50 mM); for the mixed-substrate scenarios, we used 1:1 carbon-equivalent mixture of glucose and succinate (respectively, 25 mM and 37.5 mM), glucose and citrate (respectively, 25 mM and 25 mM), glucose and acetate (respectively, 25 mM and 150 mM), or glucose and benzoate (respectively, 8.33 mM and 7.14 mM).

For each growth condition, the cells (three biological replicates) were transferred twice in the growth medium to ensure the cells were acclimated in the specific medium composition. Biomass growth was monitored by measuring the optical density at 600 nm (OD_{600}) using an Agilent Cary UV-visible spectrophotometer (Santa Clara, California). An exponential growth rate (h^{-1}) was determined via regression analysis. Measurement of cell dry weight in grams (g_{CDW}) as a function of exponential OD_{600} was obtained following lyophilization of sample aliquots (1.5-mL) using a Labconco (Kansas City, MO, USA) Freeze-Dryer System.

Table S1. Chemical speciation of Fe in terms of its equilibrium concentration, complexation with chelator EDTA or complexation with respective organic acid in the growth medium. Calculation for speciation of Fe in each experiment were computed using Visual Minteq (Version 3.1). The starting exogenous Fe concentration (~650 nM), provided as Fe^{2+} salts, was kept the same in all conditions.

		Equilibrium Fe concentration (nM)	EDTA bound Fe (nM)	Substrate bound Fe (nM)
**Intermediate Fe-limited conditions** ***(no EDTA added)***	Succinate-alone[1]	650	—	0
	Citrate-alone	5.49	—	645
	Glucose:Succinate	650	—	0
	Glucose:Citrate	10.2	—	640
	Glucose:Acetate	563	—	86.6
	Glucose:Benzoate	650	—	0
**Severe Fe-limited conditions** ***(90 µM EDTA added)***	Succinate[1]	31.1	619	0
	Malate	21	611	17.7
	Glucose:Benzoate	35.8	614	0

[1]The software has no constant for Fe^{2+} complexation by succinate.

Extracellular substrate depletion and extracellular metabolite excretion. To quantify substrate depletion by the cells, 0.5-mL of samples of cell suspensions from the three biological replicates were harvested during the different phases of cell growth and filter-centrifuged (Sigma Aldrich Spin-X 0.22 μm filters) at 9391*g* for 5 mins at 4°C (Centrifuge 5423 R, Eppendorf, Hauppauge, NY). Specifically, samples at the beginning of the lag phase, the substrate concentrations for were obtained at the beginning of the lag phase, at mid-exponential phase, and at the onset of stationary phase during growth on glucose:succinate, glucose:citrate, and glucose:acetate mixture scenarios; for the glucose:benzoate mixture conditions, samples were obtained at multiple timepoints over the entire course of cell growth phases. The supernatants were stored at 4°C until analysis for simultaneous quantification by ^{1}H nuclear magnetic resonance (NMR) using a Varian Unity INOVA 600-MHz NMR spectrometer at 25°C, as described previously[3]. Substrate assimilation was confirmed by metabolite labeling of substrate-derived carbons as detailed below.

Extracellular metabolite levels were measured by centrifuging cell suspensions and filtering the supernatants (50-μL) followed by 1:10 or 1:100 dilution with LC-MS grade water (Fisher Scientific, Pittsburgh, PA) before analysis via LC-MS. Dilution ratios are adjusted depending on the concentrations of the extracellular metabolites. For measuring the excretion rate of a metabolite, samples (three biological replicates) were harvested at several time points throughout the growth phase. The excretion rate (μmol g_{CDW}^{-1} h^{-1}) was then computed via regression analysis. All samples were stored at 4°C prior to processing via LC-MS.

Kinetic and long-term intracellular ^{13}C labeling. Labeled substrates were purchased from Cambridge Isotopes (Tewskbury, MA, USA). We tracked kinetic isotopic incorporation of [U-$^{13}C_6$]-glucose or [U-$^{13}C_4$]-succinate into intracellular metabolites to capture *in vivo* cellular fluxes into siderophore precursors[1,4]. In liquid cultures at mid-exponential-phase when half of the substrate was consumed, a matching amount of labeled substrate was added to the nonlabeled substrate remaining in the media to reach a 1:1 ratio. Samples were then taken at short time intervals (2, 5, or 15 mins) and the incorporation of ^{13}C-labeled glucose or ^{13}C-labeled succinate into metabolites was monitored using LC-MS, Data from samples taken immediately before the addition of the labeled substrate are plotted for time = 0.

We also monitored long-term intracellular incorporation of substrates by growing liquid cultures for at least two doubling times in media solutions containing [1,4-$^{13}C_2$]-succinate alone, [U$^{13}C_4$]-succinate with unlabeled glucose, [U-$^{13}C_6$]-glucose with unlabeled succinate, [U-$^{13}C_6$]-glucose with unlabeled citrate, or [U-$^{13}C_6$]-glucose with unlabeled benzoate under the different aforementioned Fe-replete and Fe-limited conditions. Samples were taken during exponential phase. Method details on sample extraction prior to LC-MS analysis are provided elsewhere[3].

Metabolomic analysis via LC-MS. Samples were analyzed by reversed-phase ion-pairing LC via ultra-high performance LC (UHPLC; Thermo Scientific DionexUltiMate 3000) coupled to a high-resolution accurate-mass mass spectrometer (MS; Thermo Scientific Q Exactive) with electrospray ionization operated in full-scan negative mode (m/z range 70-900). Details on the LC parameters, including the solvent gradient and injection volume, were reported elsewhere[5]. The following metabolites and amino acids were monitored: aspartate, ornithine, 3-phosphoglycerate, α-KG, pyruvate, fumarate, succinate, gluconate, glucose-6-phosphate, fructose-6-phosphate, fructose-1,6-bisphosphate, dihydroxyacetone, phosphoenolpyruvate, 6-phosphogluconate, citrate, malate, sedoheptulose-7-phosphate, ribose-5-phosphate and xylulose-5-phosphate. Metabolite levels and ^{13}C labeling patterns were retrieved using the Metabolomics Analysis and Visualization Engine (MAVEN) software packag[6]. For the labeled data, ^{13}C-labeled fractions were corrected for natural ^{13}C abundance. Aspartate labeling was taken as a surrogate for oxaloacetate (OAA) labeling due to the direct synthesis of aspartate from OAA[4,7].

Metabolic flux ratio analysis. To determine metabolic reprogramming of cells grown on a glycolytic substrate (glucose) and a gluconeogenic substrate (benzoate) in response to Fe deficiency, we conducted a metabolic flux ratio analysis across metabolite nodes during exponential growth of cells simultaneously on [U-$^{13}C_6$]glucose and unlabeled benzoate under Fe-replete and Fe-limited conditions. Due to their location at important junctures in the network of central carbon metabolism, we focused on the following four metabolic nodes in the metabolic network: succinate, glyceraldehyde-3-phosphate, phosphoenolpyruvate, and pyruvate. We used the experimental profiling of the intracellular metabolite labeling patterns to quantify the fractional contribution of different metabolites and pathways towards each node. We obtained the optimized flux ratios by iterative optimization with the objective of obtaining the minimum error difference between experimental and simulated labeling data[8]. An example for the equations used to simulate the labeling data for metabolite GAP is provided below:

$$GAP_0 = f_1(6\text{-}PG_0) + f_2(3\text{-}PG_0) \quad \text{(Eq. 1)}$$

$$GAP_3 = f_1(6\text{-}PG_6) + f_2(3\text{-}PG_3) \quad \text{(Eq. 2)}$$

where, f_1 and f_2 refers to the fraction of GAP origination from 6-PG and 3-PG respectively; $metabolite_0$ and $metabolite_{3,6}$ refers to the non-labeled and fully-labeled fractions of the respective metabolite. We also estimated changes in the ATP, NAD(P)H, and $UBQH_2$ yield using the resulting Fe-dependent metabolic flux ratios. When a metabolic flux contribution can involve enzymes with different co-factors, equal contribution of each enzyme was assumed.

Siderophore characterization and Quantitation. Using LC-MS-MS analysis, we followed a published procedure[9] for isolation and characterization of PVD siderophore secreted by exponentially-growing *P. putida* KT2440 grown on unlabeled glucose, unlabeled succinate, unlabeled citrate, 1:1 U-$^{13}C_6$ glucose glucose:unlabeled succinate (carbon-equivalent) mixture, and 1:1 U-$^{13}C_6$ glucose:unlabeled citrate (carbon-equivalent) mixture under intermediary Fe-limited condition. The peaks for PVD candidates in the bacterial supernatants were identified from the full scan total ion chromatogram by extracting the fragments of the 2,3-diamino-6, 7-dihydroxyquinoline present in all PVD siderophores[9–11]. The PVD structures were elucidated following collision-induced MS/MS fragmentation. Both the doubly charged ion $[M+2H]^{2+}$ and the single charge ion $[M+H]^{+}$ were used as precursors in the collision cell[9]. Following the structure elucidation, we compared the distribution of isotopologue *m/z* values of $[M+H]^{+}$ PVD ions in the secretion under the different nutrient growth conditions.

The extracellular amount of total secreted PVD siderophores was determined in pH-adjusted (pH 7) bacterial supernatants as done previously using absorbance measurements at 400 nm[4]. A PVD standard (Sigma Aldrich) isolated from a *P. fluorescens* strain was used for quantitation, based on previous LC-MS verification that structures of PVD from the different *Pseudomonas* species have the same dihydroxyquinoline-type chromophore[9]. We determined maximum yield of secreted PVD or PVD excretion rate (μmol g_{CDW}^{-1} h^{-1}) from regression analysis of time-course data taken from exponentially-growing liquid cultures with glucose alone, fructose alone, succinate alone, malate alone, or glucose with benzoate.

Mineral Dissolution experiments. Mineral dissolution experiments of three Fe-bearing minerals were conducted with both Fe-replete and Fe-limited bacterial secretions following growth on different substrates (glucose alone, succinate alone, or a mixture with glucose and benzoate). These experiments (10 mL) were performed in 50-mL propylene tubes with the following Fe (oxyhydr)oxide minerals at 1 g L^{-1}: hematite (Fe_2O_3), goethite [FeO(OH)], or magnetite (Fe_3O_4). Cell-free supernatants (from three biological replicates) were obtained at the end of exponential phase by centrifugation (~20,000 *g*, 5 min) followed by filtering. Prior to the mineral addition, all solutions were adjusted to pH 7.5. Experiments were conducted in an incubator shaker as described above for the cell culturing, and the tubes were wrapped in aluminum foil to exclude light-mediated reactions. After 100-h reaction time, samples were centrifuged, filtered and was adjusted to pH 4.5 with acetate buffer prior to analysis. The dissolved Fe concentration was measured via ICP-AES analysis, with a detection limit of 43.7 nM. Metal concentrations were blank-corrected with control experiments conducted with cell extracts without minerals.

Protein extraction. At mid-exponential phase (OD_{600} = 0.5-0.7), sample aliquots (25-mL) were harvested from biological replicates (n = 3) and centrifuged (at 4 °C). Cell pellets were washed twice with high-purity deionized (Milli-Q grade) water and store at 20 °C for further use. Prior to analysis, pellets were thawed, resuspended in 4-mL of 2.5 mM phosphate buffer (pH 7) and vortexed. Cell lysis was then conducted in a French Pressure cell press (Thermo Spectronics, Waltham MA) with double 3-min cycles at 900 psi. Following cell lysis, aliquots were centrifuged (16000g, 10-min, 4 °C), and supernatant (2 mL) was concentrated to 200-µL using a Spin-X® UF 500 Concentrator (Corning, Tewksbury, MA). Proteins were then precipitated overnight at -20 °C in 9 volumes of cold 10% trichloroacetic acid in acetone and centrifuged (16000g, 10-min, 4 °C) to form a protein pellet. The pellets were washed twice in cold acetone following centrifugation, and urea (7 M) was added to initiate protein dissolution. Dissolved pellets were then diluted in 50 mM triethylammonium bicarbonate (TEAB) buffer (pH 8). The urea contamination in the final sample was less than 1 M.

Protein concentration was determined by Bradford assay and further quantified by running a precast NOVEX 12% Tris/Glycine mini-gel (Invitrogen Carlsbad, CA) along with an *E. coli* lysate gradient (2, 5, 10, 20 µg/lane). Visualization for the SDS gel was carried out using colloidal Coomassie blue stain (Invitrogen) imaged by Typhoon 9400 scanner followed by ImageQuant TL 8.1 (GE Healthcare). Further processing was performed according to Thermo Scientific's TMT Mass Tagging Kits and Reagent protocol with minor modification[12,13]. Briefly, a total of 200 µg protein of each sample in 70-µL of 7 M urea and 50 mM TEAB was reduced [with 11 mM tris (2-carboxyethyl)phosphine), 1 h], alkylated (with 37 mM iodoacetamide, 1 h in the dark) and quenched [with 40 mM dithiothreitol (DTT)]. Following quenching, dilutions with 240-µL of 100 mM TEAB were carried out, and samples were digested (with 18 µg trypsin, 18 h, 35 °C); an additional 2 µg trypsin was then added and incubated for 4 h. The TMT 10-plex labels were reconstituted with 45-µL of anhydrous ACN prior to labeling and added with 1:2 ratio to each of the tryptic digest samples. In order to achieve TMT labeling, the samples were incubated for 1 h at room temperature. The peptides from 10 samples were mixed with each tag of TMT10-plex separately. After mixing 1-µL aliquots from each sample and desalting with SCX ziptip (Millipore, Billerica, MA), the label incorporation was checked using Orbitrap Fusion (Thermo-Fisher Scientific, San Jose, CA). Equal amounts of peptides from the 10 digested samples were then pooled together and evaporated to 200-µL and subsequently purified by solid-phase extraction on Sep-Pak Cartridges (Waters, Milford, MA) followed by drying at reduced pressure in a Speed-Vac concentrator (Savant).

Proteomics analysis using nano-scale reverse phase LC-MS. First dimensional fractionation was carried out on the above described tryptic peptides via high-pH reverse-phase chromatography using a Dionex UltiMate 3000 HPLC system (Thermo Scientific, Sunnyvale, CA) as reported previously[12,13]. Briefly, the TMT 10-plex tagged tryptic peptides were reconstituted in 20 mM ammonium formate at pH 9.5 (eluent A) and loaded onto an XTerra MS C18 column (3.5 µm, 2.1x 150 mm) from Waters, (Milford, MA). The chromatography was carried out using a gradient from 10-45% eluent B over 30-min at a flow rate 200-µL/min. Forty-eight fractions were collected at 1-min intervals, and based on the absorbance at 214 nm and with multiple fraction concatenation strategy[12], were pooled into a total of 10 fractions. Ten percent of each fraction was pooled, dried, and reconstituted in 120 µL of 2% ACN/0.5% FA for low pH nano-scale LC-MS/MS analysis. The remaining 90% of each sample was pooled into 5 fractions, dried and subjected to titanium dioxide (TiO2) enrichment using a TiO2 Mag Sepharose kit (from GE Healthcare). Specifically, the TMT 6-plex tagged tryptic peptides were reconstituted in 400 µL of binding buffer (1 M glycolic acid in 80% ACN, 5% TFA). The TiO2 slurry (75 µL) was used and incubated with the sample for 30-min at 1,800 rpm vortex. After washing the beads with washing buffer (80% acetonitrile, 1%TFA), the phosphopeptides were eluted with 100 µL of elution buffer (5% ammonium hydroxide) twice. The eluted fractions were subsequently dried and reconstituted in 25 µL of 0.5% formic acid (FA) for nano-scale LC-MS/MS analysis using an Orbitrap Fusion (Thermo-Fisher Scientific, San Jose, CA) mass spectrometer (MS) equipped with a nanospray Flex Ion Source similar to previous reports[14,15].

The MS was coupled to an UltiMate3000 RSLCnano HPLC (Thermo Scientific, Sunnyvale, CA). Specific amounts of the reconstituted fraction (2-3 µL for global proteomics fractions and 5-8 µL for enriched phosphor-fractions) was injected onto a PepMap C-18 RP nano-trap column (100 µm x 20 mm dimensions, 3-µm particle size, Dionex) at 20 µL/min flow rate for concentration and on-line desalting. Bound peptides were separated on a PepMap C-18 RP column (75 µm x 25 cm, 3.5 µm particle size) by elution at a flow rate of 300 nL/min using a gradient of 5% to 35% acetonitrile (ACN) in 0.1% formic acid. Prior to subsequent runs, the column was re-equilibrated with 5% ACN-0.1% FA for 25 min. The Orbitrap Fusion was operated in positive ion mode with the nano spray voltage set at 1.6 kV and the source temperature at 275 °C. The Fourier transform (FT), ion-trap (IT), and quadrupole mass analyzers were externally calibrated; the MS was operated in the data-dependent acquisition (DDA) mode, and the FT mass analyzer was used for survey scans to select precursor ions. Subsequently, 3 second top speed data-dependent HCD-MS/MS scans were run using a normalized collision energy of 37.5% for all precursor ions with 2-7 charges per ion. The threshold ion count was > 10,000. MS survey scans were conducted at a resolving power of 120,000 FWHM (measured at m/z 200) for all the masses in the range of m/z 400-1600 using the settings AGC = 3e5 and Max IT = 50 ms. The MS/MS scans were conducted using higher-energy collision dissociation (HCD) at 60,000 FWHM resolution for the mass range m/z 105-2000. The AGC setting, Max IT, and Q isolation window were set to 1e5, 120 ms and ±1.6 Da, respectively. The dynamic exclusion parameters were set at 1 with a 45 second exclusion duration with a ±10 ppm exclusion mass width. All data were acquired using the Xcalibur 3.0 operation software and Orbitrap Fusion Tune 2.0 (Thermo-Fisher Scientific).

Data interpretation and protein identification. Raw spectral data (MS and MS/MS) were processed and searched using Proteome Discoverer 2.2 (Thermo) with Sequest HT. Data was queried using the Pseudomonas_putidaKT2440.fasta database containing 5528 entries (downloaded June 12, 2017). The default search settings used for 10-plex TMT quantitative processing and protein identification were as follows: tryptic proteolysis, allowing two missed cleavages, with fixed carbamidomethyl modification of cysteine, fixed 10-plex TMT modifications of lysine and N-terminal amines and variable modifications of methionine oxidation and deamidation of asparagine/glutamine residues. The peptide and fragment mass tolerance values were 10 ppm and 50 mDa, respectively. The identified peptides were filtered for a maximum 1% False Discovery Rate using the Benjamini Hochberg procedure[16] incorporated in the Percolator algorithm in PD 2.2 with the peptide confidence was set to high. The TMT10-plex quantification method within the PD 2.2 software was used to calculate the reporter ratios with mass tolerance ±10 ppm without applying the isotopic correction factors. The peptide spectra containing all reporter ions were designated as quantifiable and used for peptide/protein quantitation. Summed intensity normalization was used to estimate expression ratios. Expected ratio distortion due to the co-isolation of near isobaric precursor ions was minimized by applying a co-isolation filter of 50%.

Statistical Analysis. Statistical analysis of growth, physiological, and metabolomics experiments were carried out using One-way Analysis of Variance (ANOVA) test. A Tukey analysis was subsequently used as a *post hoc* analysis. Statistically-significant difference was determined at $p \leq 0.05$. For the proteomics data, the thresholds for significant change was estimated by carrying out log2 transformations of the expression ratios from each of the data sets and consequently fitting them to 60 different statistical distributions using the program EasyFit (MathWave Technologies). The goodness of fit was determined by the Kalmogrov/Smirnov, Anderson Darling, and Chi-Squared tests[17–19]. In each case the best fit was determined to be to the four- parameter Dagum distribution[20]. The 95% confidence intervals were estimated by generating distributions randomly using the exact parameters of the experimentally derived distributions. to evaluate the difference between the means of the samples being compared, a two tailed heteroscedastic *t*-test was conducted. An expression ratio was considered to have changed significantly if the magnitude of the change was greater than what would be expected to be observed by chance with a probability of 0.05, and if its associated p-value was < 0.01.

Data availability. Source data sets are uploaded in open-source repositories for free accessibility. All MS proteomics data are deposited in the ProteomeXchange (data set identifier PXD013605).

Uploaded on the MetaboLights database[21] (study identifier MTBLS1715) are the LC-MS data along with the methodological details for the intracellular metabolite labeling experiments with *P. putida* KT2440 grown on [U-$^{13}C_6$]-glucose and unlabeled benzoate under Fe-replete, intermediate-Fe limited, Fe-limited Phase 1 and Fe-limited Phase 2 conditions. Also uploaded on Metabolites database are the following datasets obtained with *P. putida* KT2440: kinetic labeling in glucose-grown and succinate-grown Fe-replete and intermediate Fe-limited cells, long-term labeling with [1,4-$^{13}C_2$-succinate] in Fe-replete and intermediate Fe-limited cells, long-term labeling with [U-$^{13}C_4$-succinate] and unlabeled glucose in Fe-replete and intermediate Fe-limited cells, long-term labeling with [U-$^{13}C_6$-glucose] and unlabeled citrate in intermediate Fe-limited cells, and mass spectra of major pyoverdine ion in Fe-limited cells grown on glucose alone, succinate alone, citrate alone, ^{13}C-glucose:succinate, or ^{13}Cglucose:citrate. To access these files on MetaboLights: 1. Please visit http://www.ebi.ac.uk/metabolights/, 2. Navigate to the search bar on the top-right of the page, and 3. Enter study identifier MTBLS1715.

Table S2. Fold changes of protein abundance in glucose:benzoate grown *P. putida* KT2440 cells grown under Fe-limited conditions with respect to cells grown under Fe-replete conditions. Grey boxes indicate the lack of quantifiable proteins. Refer to the legends of Fig. 1 in the main text for the metabolite abbreviations. All the data obtained from biological replicates (n=3) are shown.

Reactions	ORF number	Proteins	Protein abundance (Average) (-Fe/+Fe)	(p-values)
$Gluc_{ext}$ → $Gluc_{peri}$	PP_1019	oprB-1	0.826	0.303
$Gluc_{peri}$ → Gluc	PP_1015	gtsA		
$Gluc_{peri}$ → Gluc	PP_1016	gtsB	0.62	0.011
$Gluc_{peri}$ → Gluc	PP_1017	gtsC		
$Gluc_{peri}$ → Gluc	PP_1018	gtsD		
Gluc → G6P	PP_1444	gcd	1.171	0.406
$Gluc_{peri}$ → $Glucn_{peri}$	PP_3417	gntP	0.861	0.438
$Glucn_{peri}$ → Glucn	PP_3382	PP3382	0.795	0.175
$Glucn_{peri}$ → 6-PG	PP_3384	PP3384	0.903	0.580
$Glucn_{peri}$ → $2KGlucn_{peri}$	PP_3383	PP3383		
$Glucn_{peri}$ → $2KGlucn_{peri}$	PP_3377	kguT		
$Glucn_{peri}$ → $2KGlucn_{peri}$	PP_3378	kguK	0.896	0.508
$2KGlucn_{peri}$ → 2KGlucn	PP_3376	kguD		
2-KGlucn → 2KGlucnP	PP_1011	glk	0.869	0.362
2KGlucnP → 6-PG	PP_3416	gnuk	0.797	0.190
G6P → 6-PG	PP_1024	pgl	0.709	0.077
G6P → 6-PG	PP_4042	zwfB		
G6P → 6-PG	PP_5351	zwf		
G6P → 6-PG	PP_1022	zwfA		
6-PG → KDPG	PP_1010	edd		
KDPG → Pyruvate + GAP	PP_1024	eda	0.715	0.085
GAP → 1,3-bisPG	PP_2149	gap-2	0.817	0.319
GAP → 1,3-bisPG	PP_1009	gap-1	0.781	0.214
1,3-bisPG → 3-PG	PP_4963	pgk	0.93	0.775
2-PG ↔ 3-PG	PP_5056	pgm		
2-PG ↔ PEP	PP_1612	eno		
Pyruvate → PEP	PP_1362	pykA	1.07	0.632
PEP → Pyruvate	PP_4301	pykF	0.964	0.767
PEP → Pyruvate	PP_2082	ppsA	1.022	0.820
DHAP ↔ GAP	PP_4715	tpiA		
FBP ↔ DHAP + GAP	PP_4960	fda	1.049	0.713
FBP → F6P	PP_5040	fbp		
G6P ↔ F6P	PP_1808	pgi1		
G6P ↔ F6P	PP_4701	pgi2	0.926	0.754
6-PG → Ru5P + CO_2	PP_4043	gnd		
Ru5P ↔ R5P	PP_5150	rpiA		
Ru5P ↔ Xu5P	PP_0415	rpe	0.865	0.410
Xu5P + R5P ↔ GAP + S7P	PP_4965	tktA	0.902	0.650
S7P + GAP ↔ E4P + F6P	PP_2168	tal	1.3	0.124
F6P + GAP ↔ E4P + Xu5P	PP_4965	tktA	0.902	0.650
Pyruvate → Acetyl-CoA + CO_2	PP_0339	aceE	0.973	0.966
Pyruvate → Acetyl-CoA + CO_2	PP_0338	aceF	0.981	0.999
OAA → Pyruvate + CO_2	PP_5347	accC2	0.786	0.227
Pyruvate + CO_2 → OAA	PP_5346	oadA	0.684	0.049
OAA → PEP + CO_2	PP_1389	PP1389	1.188	0.368
OAA → PEP + CO_2	PP_1505	ppc	1.248	0.219
OAA + Acetyl-CoA → Citrate	PP_4194	gltA	0.899	0.634

Cis-aconitate → Isocitrate	PP_2339	acnB	1.055	0.6887
Isocitrate → α-KG	PP_4011	PP_4011	1.942	0.0002
Isocitrate → α-KG	PP_4012	icd	0.974	0.971
α-KG → Succinyl-CoA + CO_2	PP_4189	kgdB		
α-KG → Succinyl-CoA + CO_2	PP_4188	kgdA		
α-KG → Succinyl-CoA + CO_2	PP_4187	lpdG		
α-KG → Succinyl-CoA + CO_2	PP_4404	lpdV	1.22	0.301
α-KG → Succinyl-CoA + CO_2	PP_5366	lpd	1.557	0.0131
Succinyl-CoA → Succinate	PP_4186	sucC		
Succinyl-CoA → Succinate	PP_4185	sucD		
Succinate → Fumarate	PP_4191	sdhA	0.678	0.044
Succinate → Fumarate	PP_4190	sdhB	0.581	0.004
Succinate → Fumarate	PP_4193	sdhC	0.353	4.45E-09
Fumarate → Malate	PP_0944	fumC	4.086	7.10E-15
Fumarate → Malate	PP_1755	fumC-2		
Malate + NAD(P)+ → OAA + NAD(P)H + H+	PP_0654	mdh		
Malate + a quinone → oxaloacetate + a quinol	PP_0751	mqo1	1.055	0.688
Malate + a quinone → oxaloacetate + a quinol	PP_1251	mqo2	0.984	0.986
Malate + a quinone → oxaloacetate + a quinol	PP_2925	mqo3	1.622	0.0081
Malate → Pyruvate + CO_2	PP_5085	maeB		
$Benzoate_{ext}$ → $Benzoate_{peri}$	PP_1377	pcaF	1.138	0.416
$Benzoate_{ext}$ → $Benzoate_{peri}$	PP_3951	pcaI		
$Benzoate_{peri}$ → Benzoate	PP_3952	pcaJ	1.139	0.414
Benzoate → Benz-1,2-diol	PP_1380	pcaD		
Benzoate → Benz-1,2-diol	PP_3714	catC	1.198	0.275
Benzoate → Benz-1,2-diol	PP_3715	catB	1.183	0.306
Benz-1,2-diol → Catechol + CO_2	PP_3166	catA2	1.059	0.799
Catechol → cis,cis-Muconate	PP_3713	catA	0.928	0.765
Catechol → cis,cis-Muconate	PP_3164	benD		
cis,cis-Muconate → Muconolactone	PP_3161	benA		
Muconolactone → 4-ketoadipate enol-lactone	PP_3162	benB		
4-ketoadipate enol-lactone → 3-ketoadipate	PP_3163	benC	1.037	0.760
3-ketoadipate → 3-ketoadipyl-CoA	PP_3165	benK	1.123	0.567
	PP_3168	benF		
3-ketoadipyl-CoA → Succinyl-CoA + Acetyl-CoA	PP_3167	benE-2		
Citrate → Glyoxylate + Succinate	PP_4116	aceA	0.902	0.649
Glyoxylate + Acetyl-CoA → Malate	PP_0356	glcB	1.473	0.0263

Estimation of CO_2 production. To close the carbon balance within our systems, we estimated the amount of CO_2 generated in the nine CO_2 producing metabolic reactions within substrate catabolism and central carbon metabolism. We computed these estimations for *P. putida* KT2440 cells grown on 1:1 glucose:benzoate mixture under Fe-replete, intermediate Fe-limited and severe Fe-limited conditions. For the Fe-replete condition, we calculated the CO_2 produced based on a previous metabolic flux analysis performed on the same strain, growth media and Fe condition[3]. The estimations of CO_2 produced in the intermediate Fe-limited condition and first phase of the severe Fe-limited condition was based on the intracellular labeling patterns of *P. putida* cells grown on [U-$^{13}C_6$]-glucose with unlabeled benzoate, as well as flux ratio analysis (described above) for their respective Fe conditions. For the second phase of the severe Fe-limited condition, CO_2 generated was computed based on a reported metabolic flux analysis performed on *P. putida* KT2440 cells grown on Fe-limited glucose-only media[4]. When relevant, carbon effluxes (metabolite secretion, PVD production, and biomass production) were subtracted from the model at the respective metabolite nodes. For instance, the gluconate secretion rate was reflected by the loss of gluconate from the periplasm, and subsequent reduced flux of gluconate towards 6-phosphogluconate. Additionally, the carbon consumption rate was used to obtain CO_2 produced during substrate catabolism (Benz-1,2-diol → Catechol + CO_2) (Table S2).

Table S3. List of nine CO_2-producing and CO_2-consuming metabolic reactions considered for the CO_2 efflux estimation (see Fig. 7A in the main text).

Pathway	Reactions
Pentose Phosphate Pathway	6-PG → Ru5P + CO_2
Downstream Entner-Doudoroff Pathway	Pyruvate → Acetyl-CoA + CO_2
Gluconeogenesis/Anaplerosis	OAA → Pyruvate + CO_2
	Pyruvate + CO_2 → OAA
	OAA → PEP + CO_2
	Malate → Pyruvate + CO_2
Tricarboxylic Acid Cycle	α-KG → Succinyl-CoA + CO_2
	Isocitrate → α-KG
Initial Aromatic Substrate Catabolism	Benz-1,2-diol → Catechol + CO_2

Table S4. Secretion rates of organic acid metabolites during growth of *P. putida* KT2440 on the glucose:benzoate mixture under the Fe-replete, intermediate Fe-limited, and severe Fe-limited conditions. Grey boxes indicate the lack of quantifiable metabolites in the extracellular medium. All the data obtained from biological replicates (n=3) are shown.

	**Fe-replete**	**Intermediate Fe-limited**	**Fe-limited Phase 1**	**Fe-limited Phase 2**
Gluconate (in mmol C g_{CDW}^{-1} h^{-1})	0.29 ± 0.027	1.83 ± 0.220	0.10 ± 0.03	13.9 ± 1.8
Other organic acids (µmol C g_{CDW}^{-1} h^{-1})				
Succinate	35.4 ± 4.05	32.8±3.99	12.5 ± 2.64	
Oxaloacetate		6.26±1.90	35.4 ± 13.9	
Fumarate	0.95 ± 0.20	1.34 ± 0.59		
Pyruvate		8.79 ± 3.33		
α-ketoglutarate	95.9 ± 8.16	18.5 ± 4.25		

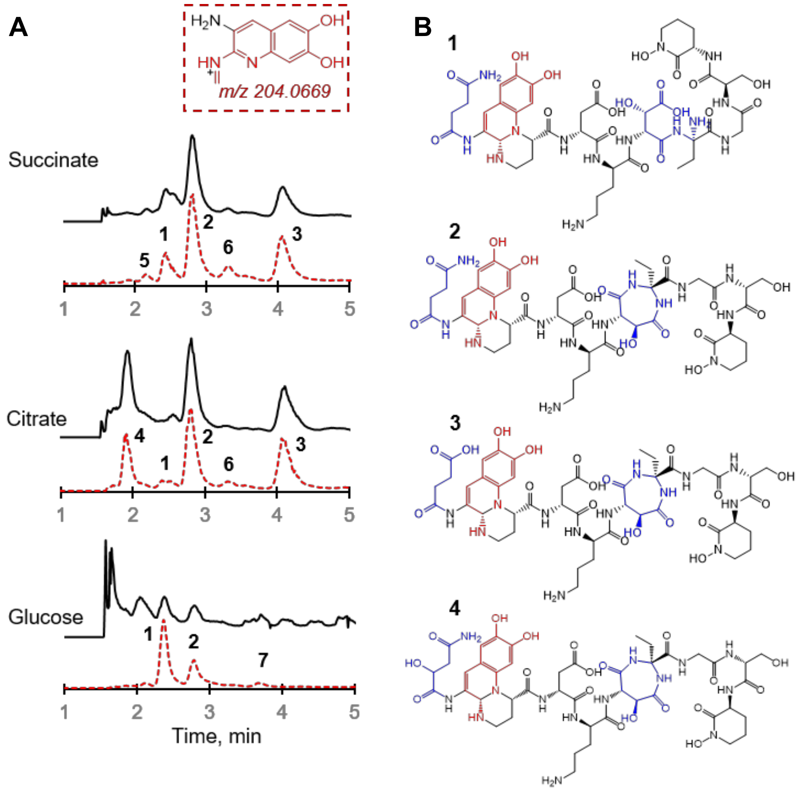

Fig. S1. A, MS full scan total ion chromatogram (black line) and extracted ion chromatogram (red line) of chromophoric fragment (m/z 204.0773) of all ion fragmentation obtained from extracellular media of *P. putida* KT2440 grown on, from top to bottom, succinate, citrate, and glucose. **B**, Chemical structures of the four major PVDs characterized; these structures are similar in terms of the metabolite precursors. In **B**, the structural differences (highlighted in blue) are the carboxylic acid or amide versions of the same side chain (succinic acid or succinimide) or linear versus cyclic version of the same amino acid (ornithine).

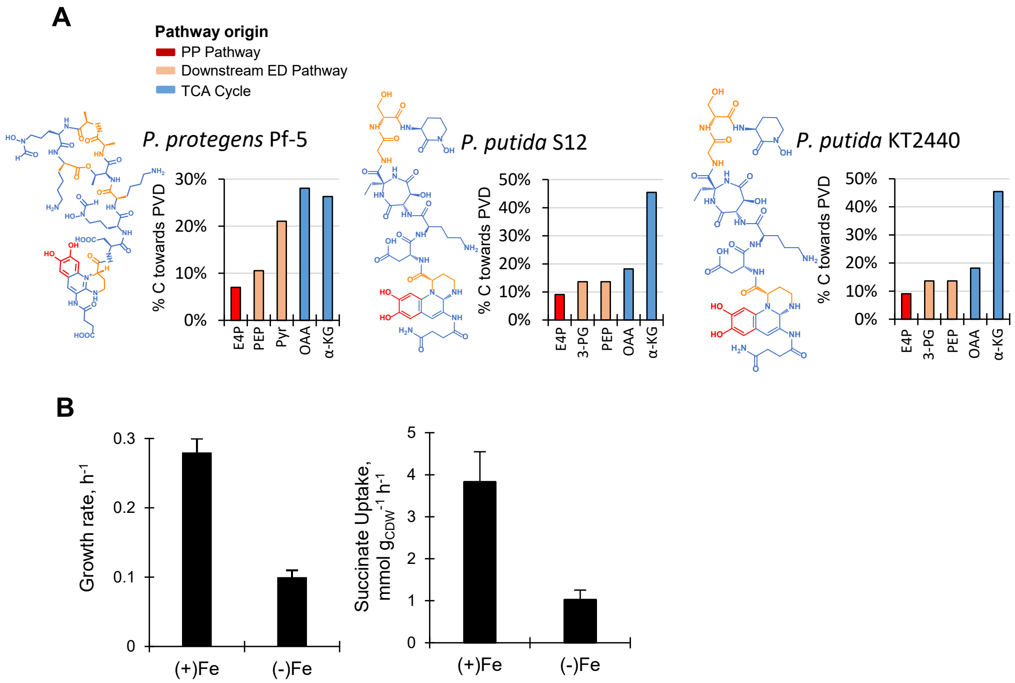

Fig. S2. A, Chemical structure of PVDs characterized in the bacterial secretion of *P. protegens* Pf-5[10,22], *P. putida* S12[22] and *P. putida* KT2440[9], and the biosynthetic investments of the respective metabolite precursors. **B**, (left) Growth rate and (right) consumption rate for succinate-grown *P. putida* KT2440 under Fe-replete [(+)Fe] and Fe-limiting [(-)Fe] conditions. Color code for **A**: pink denotes the pentose phosphate (PP) pathway, yellow represents the downstream Entner-Doudoroff pathway (ED) pathway and blue represents the tricarboxylic acid (TCA) cycle. The abbreviations are as follows: succinate, Succ; erythrose 4-phosphate, E4P; 3-phosphoglycerate, 3-PG; phosphoenolpyruvate, PEP; pyruvate, Pyr; oxaloacetate, OAA; α-ketoglutarate, α-KG. Data (average ± standard deviation) for **B** are from three biological replicates.

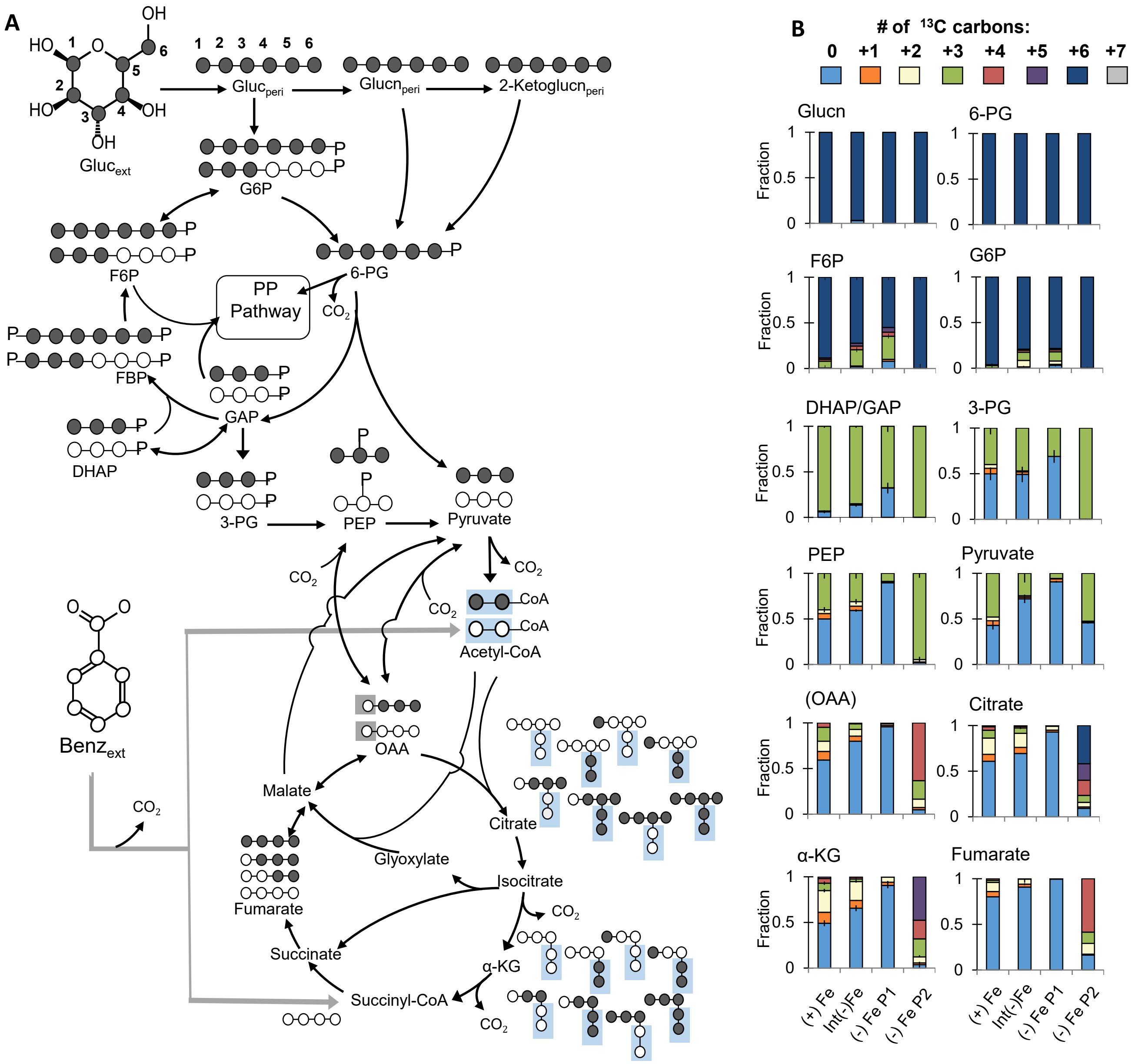

Fig. S3. Investigation of Fe-dependent substrate incorporation through the assimilation of [U-$^{13}C_6$]-glucose and unlabeled benzoate into the intracellular metabolites of *P. putida* KT2440 on, from left to right, Fe-replete [(+) Fe], intermediate-Fe limited [(Int(-)Fe], Fe-limited Phase 1 [(-)Fe P1] and Fe-limited Phase 2 [(-)Fe P2] Glucose:Benzoate mixture. Carbon mapping (**A**) and metabolite labeling (**B**) in the periplasm, the Embden–Meyerhof–Parnas (EMP) pathway, the Entner–Doudoroff (ED) pathway and the tricarboxylic acid (TCA) cycle. Labeling patterns: nonlabeled (light blue), singly labeled (orange), doubly labeled (yellow), triply labeled (green), quadruply labeled (pink), quintuply labeled (purple), sextuply labeled (dark blue) and septuply labeled (grey). Refer to the legends of Fig. 1 in the main text for the metabolite abbreviations. Isotopologue data (mean ± s.d.) were from biological replicates (n = 3).

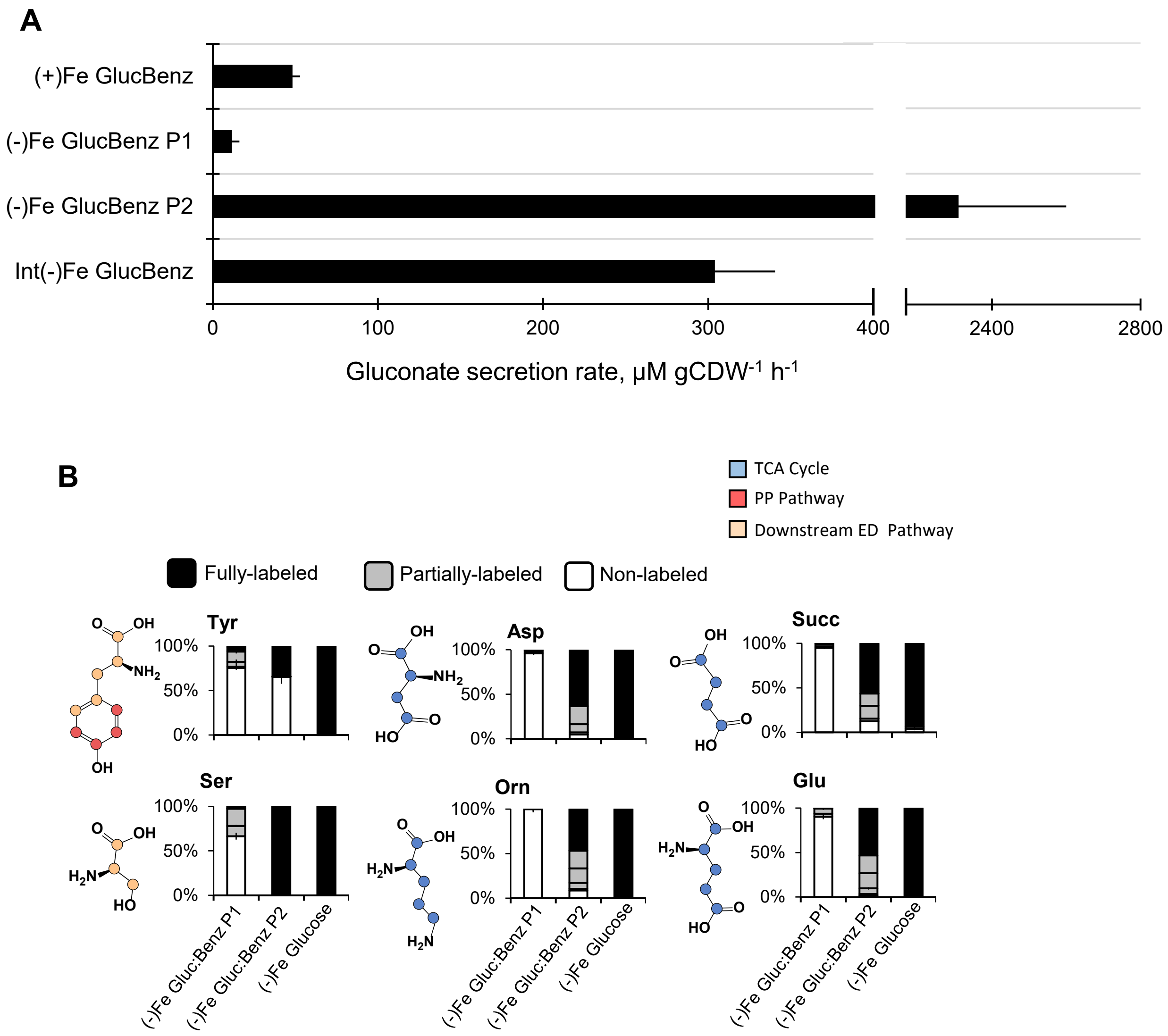

Fig. S4. A, Gluconate secretion rates of *P. putida* KT2440 cells grown on, from top to bottom, Fe-replete [(+) Fe], intermediate-Fe limited [(Int(-)Fe], Fe-limited Phase 1 [(-)Fe P1] and Fe-limited Phase 2 [(-)Fe P2] Glucose:Benzoate mixture. **B,** ^{13}C-labeled fractions of metabolites participating in the biosynthesis of PVD. Color code for pathway designation: pentose phosphate (PP) pathway in red, downstream Entner-Doudoroff pathway (ED) in light orange, tricarboxylic acid (TCA) cycle in blue. Refer to the legends of Fig. 1 for the metabolite abbreviations. All the data obtained from biological replicates (n=3) are shown.

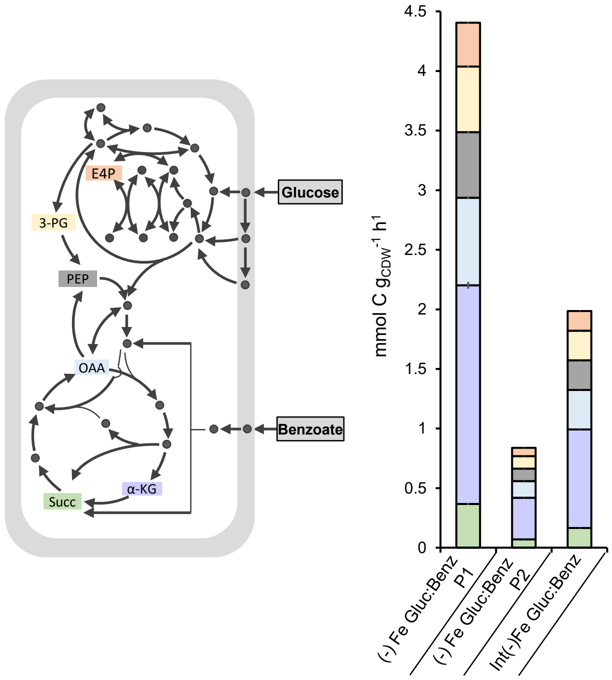

Fig. S5. PVD production rates (mmol C g_{cdw}^{-1} hr^{-1}) and predictive fluxes from metabolite precursors. PVD production rates are provided for *P. putida* KT2440 cells grown on glucose-only, glucose:benzoate Fe-limited [(−)Fe] or intermediate-Fe (Int-Fe) nutrient medium. P1 and P2 refer, respectively, to phase 1 (benzoate-only) and phase 2 (glucose-only) of the substrate consumption pattern.

SI References

1. Wushensky, J. A., Youngster, T., Mendonca, C. M. & Aristilde, L. Flux Connections Between Gluconate Pathway, Glycolysis, and Pentose–Phosphate Pathway During Carbohydrate Metabolism in *Bacillus megaterium* QM B1551. *Front. Microbiol.* **9**, (2018).
2. Kim, B. J. *et al.* Effect of iron concentration on the growth rate of *Pseudomonas syringae* and the expression of virulence factors in hrp-inducing minimal medium. *Appl. Environ. Microbiol.* **75**, 2720–6 (2009).
3. Kukurugya, M. A. *et al.* Multi-omics analysis unravels a segregated metabolic flux network that tunes co-utilization of sugar and aromatic carbons in *Pseudomonas putida*. *J. Biol. Chem.* **294**, 8464–8479 (2019).
4. Sasnow, S. S., Wei, H. & Aristilde, L. Bypasses in intracellular glucose metabolism in iron-limited *Pseudomonas putida*. *Microbiologyopen* **5**, 3–20 (2016).
5. Aristilde, L. *et al.* Glyphosate-Induced Specific and Widespread Perturbations in the Metabolome of Soil Pseudomonas Species. *Front. Environ. Sci.* **5**, 1–13 (2017).
6. Melamud, E., Vastag, L. & Rabinowitz, J. D. Metabolomic Analysis and Visualization Engine for LC−MS Data. *Anal. Chem.* **82**, 9818–9826 (2010).
7. Amador-Noguez, D. *et al.* Systems-level metabolic flux profiling elucidates a complete, bifurcated tricarboxylic acid cycle in *Clostridium acetobutylicum*. *J. Bacteriol.* **192**, 4452–61 (2010).
8. Wilkes, R. A., Mendonca, C. M. & Aristilde, L. A Cyclic Metabolic Network in *Pseudomonas protegens* Pf-5 Prioritizes the Entner-Doudoroff Pathway and Exhibits Substrate Hierarchy during Carbohydrate Co-Utilization. *Appl. Environ. Microbiol.* **85**, e02084-18 (2019).
9. Wei, H. & Aristilde, L. Structural characterization of multiple pyoverdines secreted by two *Pseudomonas* strains using liquid chromatography-high resolution tandem mass spectrometry with varying dissociation energies. *Anal. Bioanal. Chem.* **407**, 4629–4638 (2015).
10. Hartney, S. L. *et al.* Ferric-pyoverdine recognition by Fpv outer membrane proteins of *Pseudomonas protegens* Pf-5. *J. Bacteriol.* **195**, 765–76 (2013).
11. Cézard, C., Farvacques, N. & Sonnet, P. Chemistry and biology of pyoverdines, *Pseudomonas* primary siderophores. *Curr. Med. Chem.* **22**, 165–86 (2015).
12. Yang, Y. *et al.* Evaluation of Different Multidimensional LC–MS/MS Pipelines for Isobaric Tags for Relative and Absolute Quantitation (iTRAQ)-Based Proteomic Analysis of Potato Tubers in Response to Cold Storage. *J. Proteome Res.* **10**, 4647–4660 (2011).
13. Chen, J.-W. *et al.* Proteomic Comparison of Historic and Recently Emerged Hypervirulent *Clostridium difficile* Strains. *J. Proteome Res.* **12**, 1151–1161 (2013).

SI References (continued)

14. Thomas, C. J., Cleland, T. P., Zhang, S., Gundberg, C. M. & Vashishth, D. Identification and characterization of glycation adducts on osteocalcin. *Anal. Biochem.* **525**, 46–53 (2017).
15. Yang, S. *et al.* Parallel comparative proteomics and phosphoproteomics reveal that cattle myostatin regulates phosphorylation of key enzymes in glycogen metabolism and glycolysis pathway. *Oncotarget* **9**, 11352–11370 (2018).
16. Benjamini, Y. & Hochberg, Y. Controlling the False Discovery Rate: A Practical and Powerful Approach to Multiple Testing. *Source: Journal of the Royal Statistical Society. Series B (Methodological)* **57**, (1995).
17. Stephens, M. A. EDF Statistics for Goodness of Fit and Some Comparisons. *J. Am. Stat. Assoc.* **69**, 730 (1974).
18. Snedecor, G. W. & Cochran, W. G. *Statistical Methods*. (Iowa State University Press, 1989).
19. Chakravarti, I. M., Laha, R. G. & Roy, J. *Handbook of methods of applied statistics*. (John Wiley and Sons, 1967).
20. Kleiber, C. A Guide to the Dagum Distributions. in *Modeling Income Distributions and Lorenz Curves* 97–117 (Springer New York, 2008). doi:10.1007/978-0-387-72796-7_6
21. Haug, K. *et al.* MetaboLights: a resource evolving in response to the needs of its scientific community. *Nucleic Acids Res.* **48**, (2020).
22. Ye, L. *et al.* A combinatorial approach to the structure elucidation of a pyoverdine siderophore produced by a *Pseudomonas putida* isolate and the use of pyoverdine as a taxonomic marker for typing *P. putida* subspecies. *BioMetals* **26**, 561–575 (2013).

.
